# Comparison of intravascular imaging, physiological assessment and angiography for coronary revascularization in acute coronary syndrome: a systematic review and network meta-analysis

**DOI:** 10.3389/fcvm.2025.1604050

**Published:** 2025-07-21

**Authors:** Xuan-Yan Liu, Bin-Hua Ye, Xian-Dan Wu, Yue Lin, Xian Lin, Yan-Yan Li, Jing-Chao Sun

**Affiliations:** ^1^ Department of General Medicine, The First People’s Hospital of Wenling, Taizhou, Zhejiang, China; ^2^ Department of Cardiology, Taizhou Municipal Hospital, Taizhou, Zhejiang, China

**Keywords:** intravascular imaging, physiology assessment, angiography, acute coronary syndrome, coronary revascularization

## Abstract

**Background:**

The optimal percutaneous coronary intervention (PCI) technique to treat acute coronary syndrome (ACS) requires further investigation. This network meta-analysis evaluated the effects of physiological assessment and intravascular imaging techniques on the prevalence of adverse cardiac outcomes following PCIs.

**Methods:**

We reviewed PubMed, Cochrane, and EMBASE databases for the purpose of identifying all randomized control trials published up to October 30, 2024, comparing the impact of intravascular imaging, physiology assessment, or angiography techniques on outcomes. The primary outcome for this research was major adverse cardiovascular events (MACE) occurrences. Each PCI strategy was ranked *as per* the risk ratio (RR) at the 95% confidence interval (95% CI) for developing MACE.

**Results:**

Twenty-eight RCTs with 18,221 patients were identified. Compared with angiography, intravascular ultrasound (IVUS)- (RR: 0.62; 95%CI: 0.46–0.85) and fractional flow reserve (FFR)-guided PCI (RR: 0.62; 95%CI: 0.46–0.85) reduced the risk of MACE. Patients who received quantitative flow ratio (QFR)-guided PCI experienced lower all-cause mortality (RR: 0.25; 95%CI: 0.07–0.92) vs. those receiving angiography. Similarly, the RR decreased to 0.64 after using FFR-guided PCI vs. angiographic procedures (95% CI: 0.44–0.91). Compared to angiography, the subgroup analysis showed inconsistent results for IVUS-guided PCI in preventing MACE for both the optimization (RR: 0.60; 95%CI: 0.49–0.74) and decision-making (RR: 0.55; 95%CI: 0.05–6.18). The likelihood of developing MACE was lower for FFR-guided CR than for angiography-guide culprit-only PCIs (RR-0.72; 95%CI: 0.53–0.97), as confirmed by sensitivity assessment results. The research unveiled no statistically significant differences between FFR-guided culprit-only PCIs and culprit-only PCIs or angiography-guided CR.

**Conclusion:**

IVUS- and FFR-guided PCI lowers the MACE risk in patients with ACS. In addition, IVUS achieved the best results in ACS patients undergoing PCI.

**Systematic Review Registration:**

INPLASY (inplasy.com), INPLASY202420092.

## Introduction

The high prevalence of coronary artery disease (CAD) presents a considerable worldwide health burden, contributing to significant mortality and morbidity rates while exacerbating economic strain globally (1, 2). Coronary angiography is essential both for the diagnosis of CAD and for guiding revascularization (3). Despite its widespread use, the visual assessment of plaques on coronary angiography is subjective and cannot be used to reliably assess the function and impact of plaque burden on the coronary lesions (4). Several physiological assessment tools and advanced imaging techniques, such as fractional flow reserve (FFR), optical coherence tomography (OCT), and intravascular ultrasound (IVUS) could provide valuable information into ischemia-causing lesions and plaque composition (5). The current clinical evidence shows that integrating intravascular imaging and physiological assessment technologies with coronary angiography could improve diagnostic accuracy and outcomes in percutaneous coronary interventions (PCIs). Moreover, in cases of intermediate stenosis, these tools can simplify the selection of the optimal PCI technique (6, 7). However, the effect of imaging- and physiology-guided revascularization on the likelihood of developing adverse reactions in cases of acute coronary syndrome (ACS) remains unclear.

The network meta-analysis by Iannaccone et al. compared outcome data of four PCI techniques including coronary angiography, FFR, IVUS, and OCT, and the meta-regression analysis found that these techniques could improve outcomes in ACS patients (8). The subgroup analysis of a recently network meta-analysis demonstrated that the guiding of PCI for ACS patients with intravascular imaging and functional assessment is superior to using angiography alone (9). However, the outcomes of these techniques on the ACS group were derived from studies where the majority, but not necessarily all, of the patients had ACS. In addition, the impact of various intravascular imaging or physiological-based strategies on the outcomes of the PCI procedure has not been well established. These limitations may limit the generalization of the conclusions. Therefore, the benefits of physiology- and imaging-guided revascularization in ACS patients are not well established. This network meta-analysis aimed at evaluating the adverse events of various different imaging- and physiology-guided angiography techniques commonly used for coronary revascularization in ACS patients.

## Methods

We followed the Preferred Reporting Items for Systematic Reviews and Meta-Analyses (PRISMA) guidelines (10) to perform this study, which was also registered at The International Database to Register Systematic Reviews (INSPLASY) with reference number INPLASY202420092.

### Ethical considerations

Since this study depended on published studies and the data extracted from them or their Supplementary Material, no ethical approval or informed consent was needed.

### Search strategy

An electronic database search across PubMed, Cochrane, and EMBASE yielded all eligible randomized controlled trials (RCTs) published up to 30 October 2024 using the keywords “acute coronary syndrome,” “intravascular imaging,” “physiology assessment,” and “coronary revascularization” (Supplementary Table 1). We also examined references from all qualified studies to uncover additional studies.

### Selection criteria

Patients with any form of ACS, including unstable angina, T-segment elevation myocardial infarction (STEMI), and non-STEMI (NSTEMI) were qualified for this study. Only studies comparing angiography with physiological assessment or intravascular imaging vs. physiology assessment, and those that reported composite of clinical cardiovascular outcomes or major adverse cardiovascular events (MACE) were included. Papers with duplicated data from the same population that had an extended follow-up duration and comprehensive information were included. All eligible studies were reviewed by 2 researchers. Any differences in opinion between the two researchers were reviewed and an additional expert was consulted if necessary.

### Data collection

Two researchers extracted and appraised the data individually. The study characteristics and randomization technique, patient characteristics (age and gender), clinical presentation, revascularization strategies, the purpose for the intravascular imaging guidance and physiology assessment, cut-off for stent implantation, the clinical outcomes, and study follow-up duration were extracted.

### Outcomes

MACE was defined as the primary outcome measure while all-cause mortality, cardiac mortality, the number of repeat revascularizations, incidence of myocardial infarction (MI), and stent thrombosis were identified as secondary outcomes (Supplementary Table 2).

### Bias evaluation

We used the revised Cochrane risk-of-bias tool (ROB2) for assessing bias by evaluating the following five items: randomization process, missing outcome data, deviations from intended interventions, selection of the reported result, and measurement of the outcome (11). Two researchers independently categorized the studies based on the ROB2 criteria as low, some concern, or high risk.

### Data analysis

The data were analyzed with the Stata version 17 software as follows. The random-effects model was utilized to compare the risk ratios (RR) and 95% confidence intervals (95%CIs) between various angiography techniques. A continuity correction of 0.5 was added to the analysis for outcomes with zero events in any group (12). The I-squared (I²) value was calculated to determine heterogeneity. Values under 25% indicate minimal heterogeneity, 25%–50% moderate heterogeneity, and above 50% substantial heterogeneity (13). We used comparison-adjusted funnel plots to evaluate publication bias and design-by-treatment interaction model to evaluate the network-wide inconsistency (14). We checked direct and indirect evidence consistency by applying the node-splitting method to evaluate any local inconsistencies in network closed loops. The ranking of each intervention node and its relative effectiveness was calculated using cumulative probabilities as determined by surface under the cumulative ranking curve (SUCRA) values. Additionally, Visual representation of results was achieved by use of cumulative ranking plots.

### Subgroup analysis

The treatment arms were divided according to the purpose of the physiological assessment and the intravascular imaging guidance method. The studies were categorized as a decision-making trial or a PCI intervention optimization trial. The two treatments were analyzed in a separate network meta-analysis.

### Sensitivity analysis

To address any potential discrepancies between complete revascularization (CR) and culprit-only PCI, an additional sensitivity analysis was conducted by reclassifying each treatment arm into CR or culprit-only PCI. We then recalculated the pooled RR and SUCRA values for all outcomes and generated the corresponding cumulative rankograms.

## Results

### Search results

Twenty-eight RCTs were eligible for this study (Supplementary Figure 1) (15–42). The RCTs included 18,221 patients (range 63–3,505 per trial) with ACS. These trials compared a total of six interventions; angiography, FFR, IVUS, OCT, quantitative flow ratio (QFR), and optical frequency domain imaging (OFDI). The average follow-up duration varied from 6 months to 5 years. Eight RCTs (20, 25, 27, 31, 32, 34, 35, 39) conducted subgroup analysis based on ACS or non-ACS cohorts. The results of these subgroup analyses were also included. Ten trials involved patients with ACS, nine focused on patients with STEMI, five included those with NSTEMI, three targeted individuals with MI, and one trial included cases with NSTEMI or unstable angina. Supplementary Table 3 outlines the baseline characteristics of the patient.

### Bias evaluation

Inadequate allocation concealment (*n* = 16), lack of blinding (*n* = 5), and missing outcome data (*n* = 7) were identified as the most common causes of bias (Supplementary Figure 2). Eight trials were classified as low risk, fourteen studies were identified as some concern, and six were categorized as high risk. The visual funnel plot analysis revealed no publication bias for MACE, all-cause and cardiac mortality, and MI. However, asymmetrical funnel plots were noted for repeat revascularization and stent thrombosis, indicating potential publication bias for these categories (Supplementary Figures 3–8).

### Primary outcome

**Figure 1:**
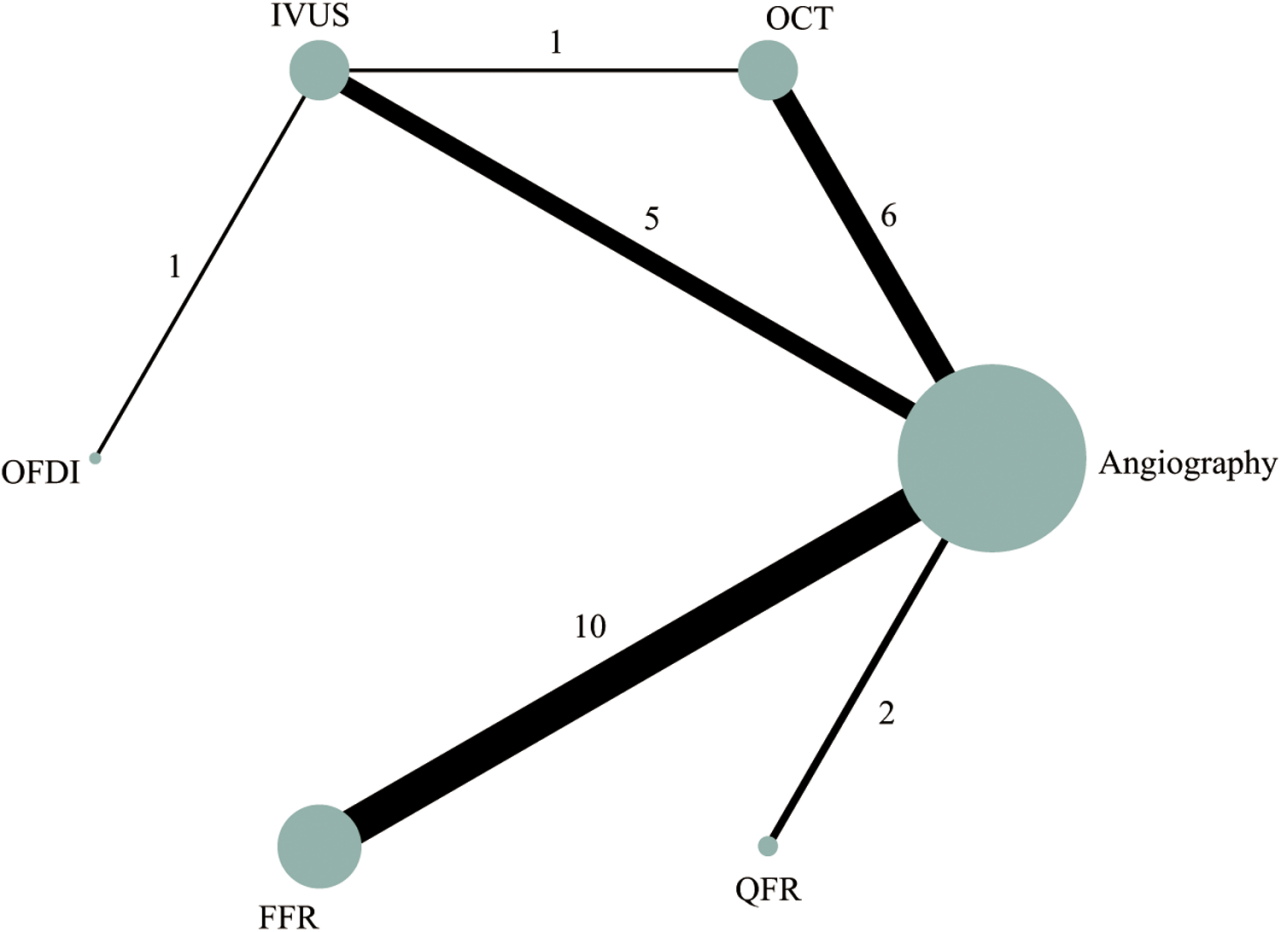
Network plot of intravascular imaging-guided, physiology-guided, and angiography-guided PCI for MACE.

**Figure 2:**
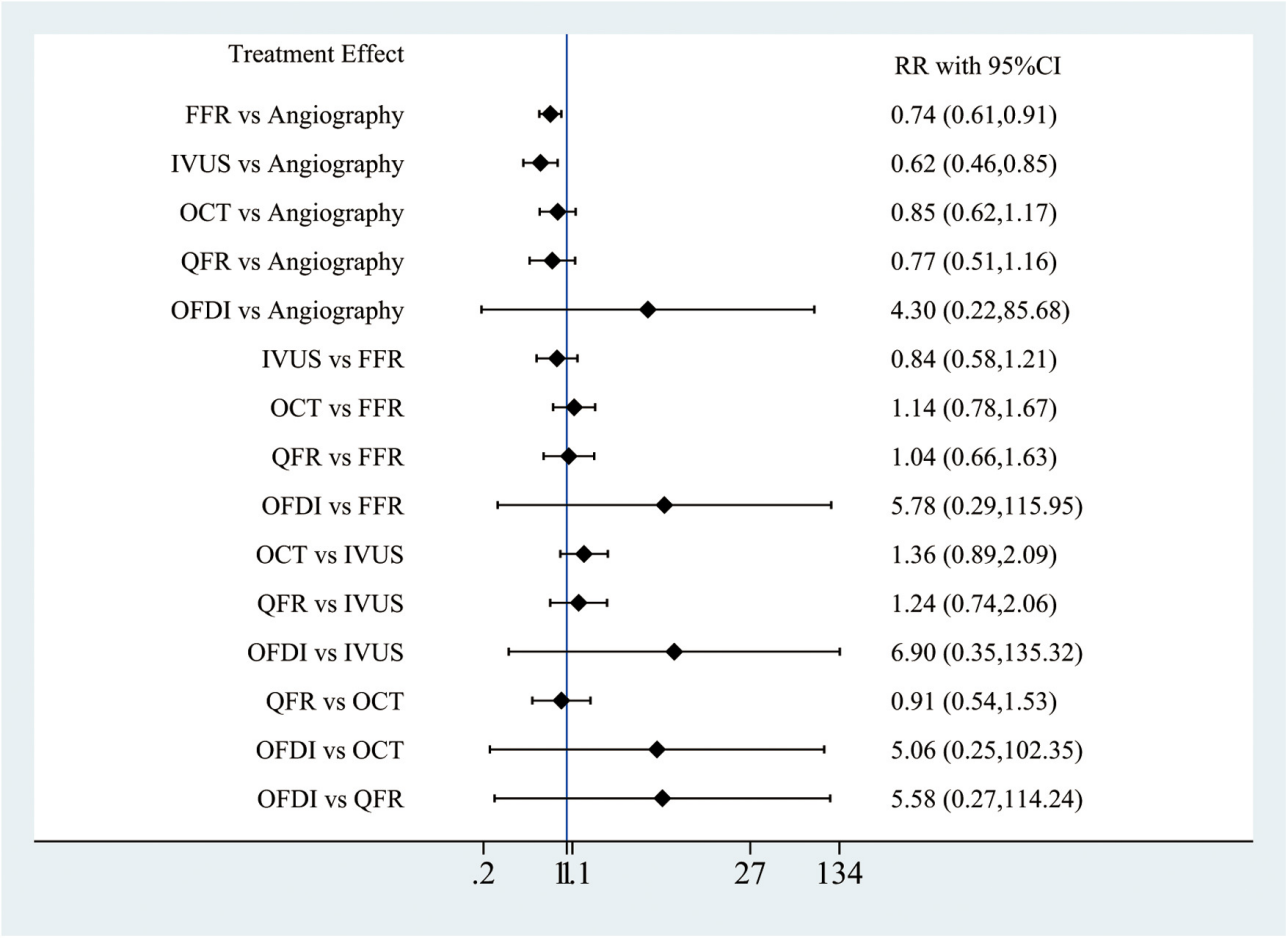
Network meta-analysis for MACE.

**Figure 3:**
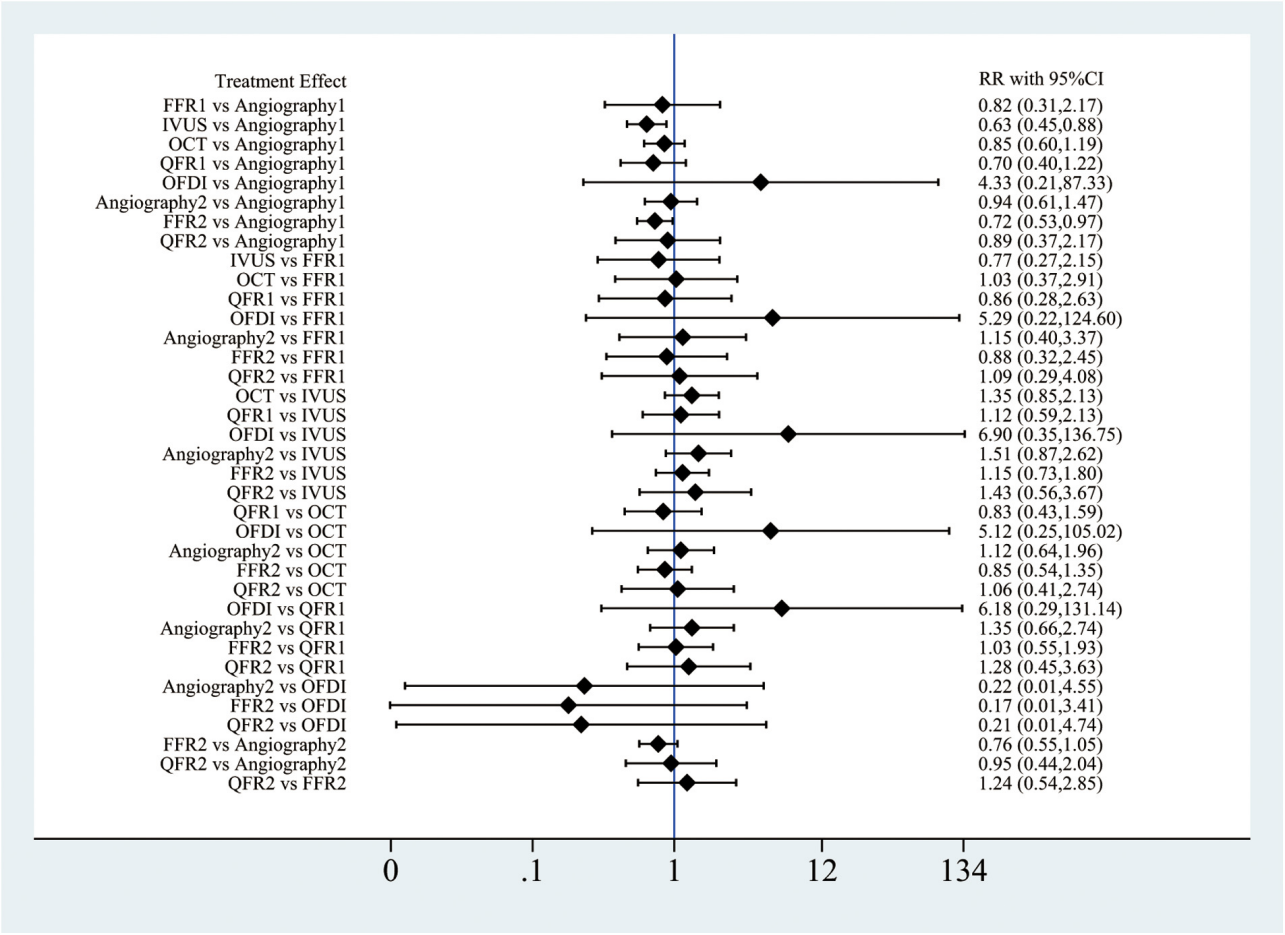
Rankogram of the six strategies for MACE.

Out of the 28 RCTs, 25 (*n* = 17,720) were incorporated in the MACE network meta-analysis (Figure 1). The closed-loop evaluation did not reveal any global or local inconsistency (*P* > 0.05). High heterogeneity was observed for studies comparing FFR and angiography. No substantial heterogeneity was found for all other comparators (Supplementary Table 4). The forest plot showed that compared with angiography both IVUS- (RR: 0.62; 95%CI 0.46–0.85) and FFR-guided PCIs (RR: 0.62; 95%CI: 0.46–0.85) were associated with a lower MACE incidence (Figure 2). In addition, there was a reduction trend in MACE for OCT (RR: 0.85; 95%CI: 0.62–1.17) and QFR (RR: 0.77; 95%CI: 0.51–1.16) compared with angiography. However, no significant difference emerged between any of the intravascular imaging and physiological strategies. The probability analysis ranked IVUS-guided PCI as the most effective strategy in reducing MACE (SUCRA 88.6%) followed by FFR-guided PCI (SUCRA 67.3%), QFR-guided PCI (SUCRA 60%), OCT-guided PCI (SUCRA 47.8%), angiography (SUCRA 22%) and OFDI-guided PCI (SUCRA 14.3%) (Figure 3).

### Secondary outcomes

The secondary outcome network plots are highlighted in Supplementary Figures 9–13. The heterogeneity between studies for each secondary outcome was calculated as shown in Supplementary Table 4. High heterogeneity was found when comparing FFR-guided PCI with angiography for the repeat revascularization and stent thrombosis outcomes, but no substantial heterogeneity was detected in other outcomes. PCIs guided by QFR led to decreased all-cause mortality vs. angiography (RR: 0.25; 95%CI: 0.07–0.92, Supplementary Figure 14) and FFR-guided PCI (RR: 0.26; 95%CI: 0.07–0.96) (Supplementary Figure 14). In addition, compared with angiography, FFR-guided PCI was associated with a lower risk of repeat revascularization (RR: 0.64; 95%CI: 0.44–0.91, Supplementary Figure 17). No significant difference between the six techniques was noted for all other secondary outcomes (Supplementary Figure 14–18). In the cumulative rankograms, QFR-guided PCI was identified as the best intervention in reducing all-cause mortality (SUCRA 85.3%, Supplementary Figure 19) and repeat revascularization (SUCRA 81.8%, Supplementary Figure 22). IVUS-guided PCI was identified as the best approach to prevent cardiac mortality (SUCRA 75.1%), MI (SUCRA 82.6%), and stent thrombosis (SUCRA 66.6%) Supplementary Figures 20–23).

### Subgroup analysis

A total of 14 RCTs were classified as decision-making or optimization trials. The revascularization strategies varied in the two MACE subgroups. FFR- and QFR-guided PCI was only used for decision-making purposes, whereas OCT- and OFDI-guided PCI were used solely to optimize the PCI procedure. IVUS-guided PCI helped lower the RR for MACE (RR: 0.60; 95%CI: 0.49–0.74, Table 1), cardiac mortality (RR: 0.45; 95%CI: 0.21–0.98, Supplementary Table 6), and MI (RR: 0.64; 95%CI: 0.45–0.93, Supplementary Table 7) in optimization subgroup when compared with the angiography. The outcomes between angiography and IVUS-guided PCI in the decision-making subgroup did not differ significantly. The subgroup analysis results for all other interventions for all outcome measures revealed similar results as those reported in the main analysis (Table 1 and Supplementary Tables 5–9).

**Table 1:** Network meta-analysis for MACE in decision-making or optimization cohorts.

Purpose	Decision-making					
Optimization	**OFDI**	NA	NA	NA	NA	NA
	NA	**QFR**	NA	1.41 (0.12,16.41)	1.05 (0.63,1.73)	0.78 (0.49,1.23)
	4.73 (0.25,91.33)	NA	**OCT**	NA	NA	NA
	6.90 (0.36,131.17)	NA	1.46 (1.08,1.97)	**IVUS**	0.74 (0.07,8.39)	0.55 (0.05,6.18)
	NA	NA	NA	NA	**FFR**	0.74 (0.60,0.92)
	4.15 (0.22,79.45)	NA	0.88 (0.69,1.11)	0.60 (0.49,0.74)	NA	**Angiography**

The bold text means intervention strategies.

### Sensitivity analysis

**Figure 4:**
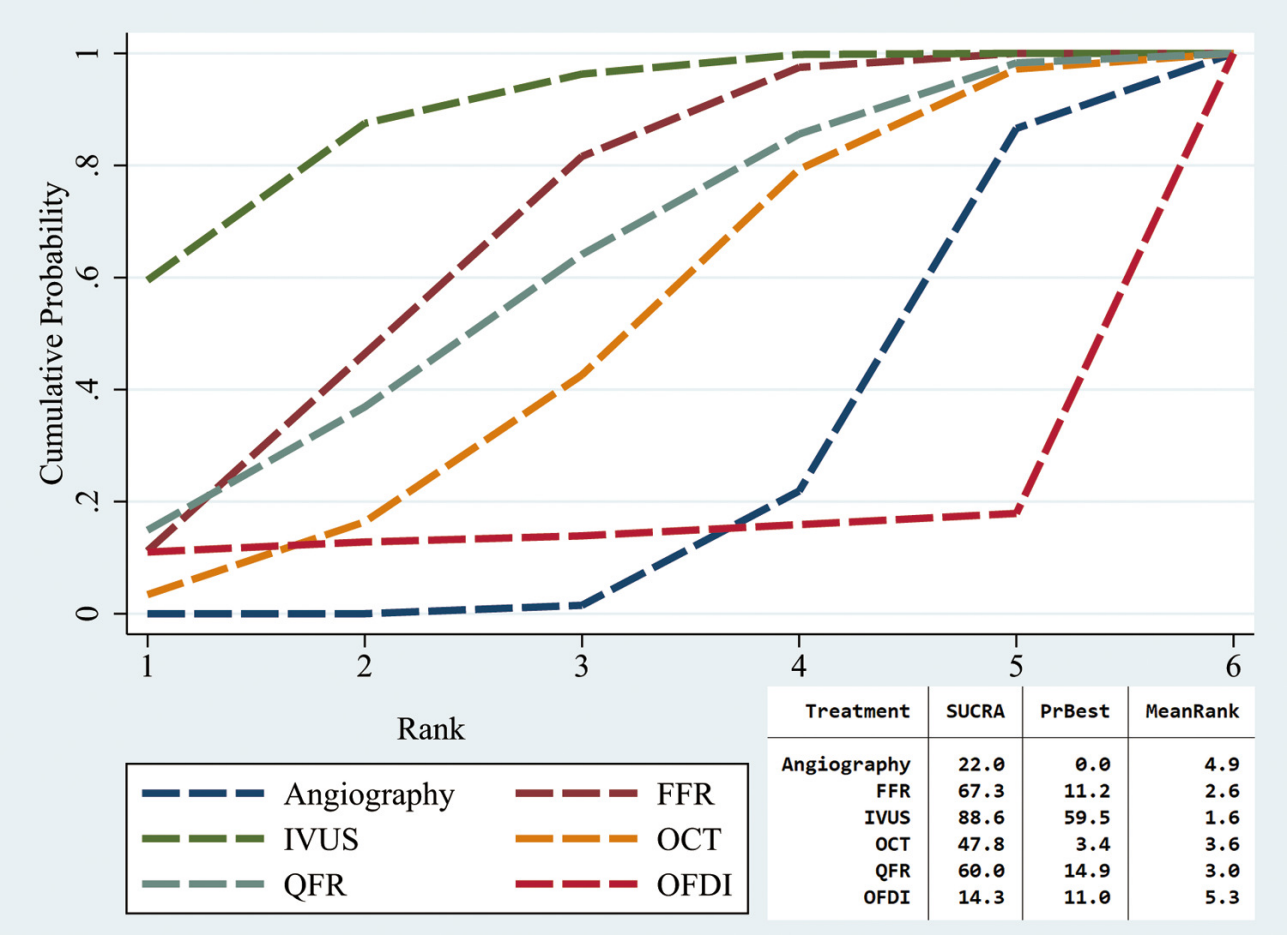
Network meta-analysis for MACE in sensitivity analysis. Angiography1, angiography-guided CR; Angiography2, Angiography-guided culprit-only PCI; FFR1, FFR-guided CR; FFR2, FFR-guided culprit-only PCI; QFR1, QFR-guided CR; QFR2, QFR-guided culprit-only PCI.

Three interventions (angiography, FFR, and QFR) made use of culprit-only PCI and CR and were included in the sensitivity analysis. Compared with angiography, both IVUS-guided culprit-only PCI (RR: 0.63; 95%CI: 0.45–0.88) and FFR-guided CR (RR: 0.72; 95%CI: 0.53–0.97, Figure 4) had a lower MACE risk. Patients receiving angiography-guided CR experienced greater likelihood of death from all causes (RR: 2.64; 95%CI: 1.11–6.27 and cardiac conditions (RR: 5.64; 95%CI: 1.46–21.75) vs. IVUS-guided culprit-only PCI. The rate of all-cause mortality turned out higher for patients receiving angiography-guided CR than for those receiving QFR-guided CR (RR: 3.97; 95%CI: 1.08–14.54) (Supplementary Figures 24, 25). FFR-guided CR demonstrated lower cardiac mortality rates vs. angiography-guided CR (RR: 0.31; 95%CI: 0.11–0.88) (Supplementary Figure 25). Furthermore, angiography-guided CR produced fewer repeat revascularization events than culprit-only PCIs guided by angiography (RR: 0.57; 95%CI: 0.37–0.88, Supplementary Figure 27). For stent thrombosis, FFR-guided culprit-only PCI showed higher risks than angiography-guided culprit-only PCI, IVUS-guided culprit-only PCI, and FFR-guided CR (Supplementary Figure 28). The other results did not differ significantly between all other revascularization strategies (Figure 4 and Supplementary Figure 24–28). The cumulative rankograms showed that IVUS-guided culprit-only PCI had the lowest MACE risk (SUCRA 81.5%, Supplementary Figures 29–34).

## Discussion

The impact of physiology- and imaging-guided revascularization techniques on the incidence of MACE in ACS patients remains unclear. While several meta-analyses have evaluated these techniques, they often included mixed populations, limiting their applicability to ACS patients. In contrast, our study specifically focuses on ACS, providing more clinically relevant insights. Additionally, subgroup and sensitivity analyses were implemented to further assess the effectiveness of intravascular imaging and physiological assessment across different revascularization strategies (CR vs. culprit-only PCI) and intervention purposes (decision-making vs. optimization).

When PCI procedures integrated IVUS and FFR, MACE occurrence decreased vs. standard angiography. Analysis using QFR as a guidance method for PCI procedures revealed lower RR of all-cause mortality than angiography and FFR-guided PCI. Additionally, the use of FFR led to fewer cases of repeat revascularization vs. angiography. However, different revascularization methods showed similar results regarding cardiac mortality, MI, or stent thrombosis. Furthermore, IVUS-guided PCI was ranked the most effective for reducing MACE, cardiac mortality, MI, and stent thrombosis, while QFR-guided PCI ranked highest for lowering all-cause mortality and repeat revascularization. Despite these findings, the advantages of performing QFR-guided PCI remain uncertain due to restricted trial inclusion.

Another Bayesian network meta-analysis evaluated the effectiveness of intravascular imaging-guided PCI vs. angiography (43). The study outcomes demonstrated that all investigated intravascular imaging interventions (IVUS and OCT/OFDI) reduced MACE incidence compared to angiography. A recent network meta-analysis evaluating the outcomes of RCTs comparing intravascular image-guided PCI with angiography also found similar results (44). Our analysis of IVUS on MACE risk was consistent with the results observed in previously published meta-analyses (43, 44). Nevertheless, no significant effect on MACE was observed for OCT or OFDI in the present analysis. Besides, our study only evaluated the revascularization strategies for patients with ACS. The results of our meta-analysis on IVUS align with current guidelines, which recommend intravascular imaging as a valuable tool for assisting coronary stent implantation (45). Nevertheless, results from our subgroup analysis demonstrated that IVUS-guided PCI optimization during PCI procedures decreased MACE risk levels than angiography alone, but with no significant difference was found in patients receiving IVUS for decision-making. This discrepancy was attributed to the different situations IVUS used for optimization or decision-making. IVUS is widely recommended for optimizing coronary stent implantation since it provides detailed visualization of the lumen and vessel wall. Moreover, the assessment of lesion length and external elastic lamina diameter through IVUS enables physicians to choose proper stent sizes and detect stent under expansion, malposition, tissue protrusion, edge dissection, and intramural hematoma following the PCI (46). IVUS is also commonly used as a diagnostic tool for ACS patients without significant coronary obstruction on angiography or in cases where the culprit lesion remains unclear (1). However, the RCT by Wang et al. (21) evaluated in our study, used IVUS for decision-making and only included STEMI patients. These patients tend to have severe coronary stenosis and often need stent implantation. Therefore, the subgroup analysis results regarding IVUS-guided PCI for decision-making should be interpreted with caution.

Our results indicate that relative to angiography, the implementation of FFR-guided PCI presents a significantly decrease the likelihood of developing MACE. However, the sensitivity analysis for the CR and culprit-only PCI demonstrated that the cardiovascular benefits of FFR-guided PCI were mainly driven by the effect of FFR-guided CR. Consistent with previous studies, no difference in the MACE morbidity was noted between the FFR-guided culprit-only PCI and angiography-guided CR or culprit-only PCI in our study (47). The FFR findings are in line with the recommendations of the European Society of Cardiology and American College of Cardiology guidelines (1, 45), which recommended FFR for angiographically intermediate stenoses in ACS patients with stable CAD or mild non-infarction-related artery (IRA) stenoses to assess the hemodynamic significance of the culprit or non-culprit lesion measurement. Accordingly, we assume that the MACE risk reduction in FFR-guided CR observed in our study was mainly attributed to the clinical cardiovascular outcome benefits generated by the revascularization of non-culprit vessels in ACS with multivessel disease.

Kuno et al. compared ACS and non-ACS patients separately and found that although intravascular-imaging-guided PCI lowered the likelihood of developing MACE and other adverse events vs. angiography, no significant difference was found in the risk of developing adverse events in patients with physiology-guided PCI (9). However, this study did not examine the effects of specific intravascular imaging or physiology assessment techniques. A similar meta-analysis of RCTs evaluated the effects of IVUS-, FFR-, and OCT-guided PCI, as well as angiography, on MACE risk and found that IVUS-guided PCI was superior to angiography, while FFR-guided PCI slightly reduced the risks of adverse events following PCI (8). Additionally, the MACE reduction rate showed a direct positive correlation with the number of ACS patients who received PCIs under IVUS and FFR guidance through meta-regression analysis. Our network meta-analysis findings for IVUS-guided PCI were consistent with these studies; however, the outcomes for FFR-guided PCI differed. Notably, three of the RCTs (20, 48, 49) included in Iannaccone et al. (8) were derived from the same trial with varying follow-up durations. Furthermore, the imprecise classification of intervention strategies for FFR and angiography may have contributed to discrepancies between our analysis and prior studies.

Our findings highlight the cardiovascular benefits of IVUS-guided PCI for optimizing stent implantation and FFR-guided CR in ACS patients with multivessel disease. These results further support the application of IVUS and FFR in ACS patients. Additional large-scale trials with rigorous study designs are essential to confirm these results and establish definitive clinical guidelines provide definitive conclusions.

### Limitations

Due to the lack of trials comparing intravascular imaging and physiology assessment, the findings of this study are based on indirect estimates. Additionally, limited overlap between intervention strategies in the two subgroups led to inadequate comparisons of each intervention's effect for different purposes. Variability in the definitions of MACE and repeat revascularization across trials may have led to the introduction of bias. Furthermore, due to the limited availability of patient data, we could not analyze the temporal relationships between intravascular imaging and physiology assessment on clinical outcomes. In addition, we could not evaluate the effect of clinical and lesion type characteristics on the incidence of adverse events for each of the imaging and physiological procedures.

## Conclusion

The application of IVUS- and FFR-guided PCI could improve outcomes and reduce the incidence of MACE than angiography. IVUS-guided PCI yielded the optimal results in lowering the risk of MACE, cardiac mortality, stent thrombosis, and MI while QFR-guided PCI ranked as the best modality for lowering all-cause mortality and repeat revascularization. Advanced intravascular imaging and physiological assessment exhibit clear benefits in optimizing PCI outcomes.

## Data Availability

The original contributions presented in the study are included in the article/Supplementary Material, further inquiries can be directed to the corresponding author.
