# Supplementary material for: Comparison of intravascular imaging, physiological assessment and angiography for coronary revascularization in acute coronary syndrome: a systematic review and network meta-analysis
Source: Front Cardiovasc Med. 2025 Jul 21;12:1604050. doi: 10.3389/fcvm.2025.1604050 (PMC12319024; doi:10.3389/fcvm.2025.1604050)
Supplement: Supplementary file 1 [file Datasheet1.docx]

**Supplementary materials**

[Supplementary Figure 1. Flow diagram of the network meta-analysis. 3](#_Toc191670354)

[Supplementary Figure 2. Risk of bias assessment in the randomized parallel-arm studies. 4](#_Toc191670355)

[Supplementary Figure 3. Comparison-adjusted funnel plots for MACE. 5](#_Toc191670356)

[Supplementary Figure 4. Comparison-adjusted funnel plots for all-cause mortality. 6](#_Toc191670357)

[Supplementary Figure 6. Comparison-adjusted funnel plots for MI. 8](#_Toc191670358)

[Supplementary Figure 8. Comparison-adjusted funnel plots for stent thrombosis. 10](#_Toc191670359)

[Supplementary Figure 9. Network plot of intravascular imaging-guided, physiology-guided, and angiography-guided PCI for all-cause mortality. 11](#_Toc191670360)

[Supplementary Figure 10. Network plot of intravascular imaging-guided, physiology-guided, and angiography-guided PCI for cardiac mortality. 12](#_Toc191670361)

[Supplementary Figure 11. Network plot of intravascular imaging-guided, physiology-guided, and angiography-guided PCI for MI. 13](#_Toc191670362)

[Supplementary Figure 12. Network plot of intravascular imaging-guided, physiology-guided, and angiography-guided PCI for repeat revascularization. 14](#_Toc191670363)

[Supplementary Figure 13. Network plot of intravascular imaging-guided, physiology-guided, and angiography-guided PCI for stent thrombosis. 15](#_Toc191670364)

[Supplementary Figure 14. Network meta-analysis for all-cause mortality. 16](#_Toc191670365)

[Supplementary Figure 15. Network meta-analysis for cardiac mortality. 17](#_Toc191670366)

[Supplementary Figure 16. Network meta-analysis for MI. 18](#_Toc191670367)

[Supplementary Figure 17. Network meta-analysis for repeat revascularization. 19](#_Toc191670368)

[Supplementary Figure 18. Network meta-analysis for stent thrombosis. 20](#_Toc191670369)

[Supplementary Figure 19. Rankogram of the six strategies for all-cause mortality. 21](#_Toc191670370)

[Supplementary Figure 20. Rankogram of the five strategies for cardiac mortality. 22](#_Toc191670371)

[Supplementary Figure 21. Rankogram of the five strategies for MI. 23](#_Toc191670372)

[Supplementary Figure 22. Rankogram of the six strategies for repeat revascularization. 24](#_Toc191670373)

[Supplementary Figure 23. Rankogram of the five strategies for stent thrombosis. 25](#_Toc191670374)

[Supplementary Figure 24. Network meta-analysis for all-cause mortality in sensitivity analysis. 26](#_Toc191670375)

[Supplementary Figure 25. Network meta-analysis for cardiac mortality in sensitivity analysis. 27](#_Toc191670376)

[Supplementary Figure 26. Network meta-analysis for MI in sensitivity analysis. 28](#_Toc191670377)

[Supplementary Figure 27. Network meta-analysis for repeat revascularization in sensitivity analysis. 29](#_Toc191670378)

[Supplementary Figure 28. Network meta-analysis for stent thrombosis in sensitivity analysis. 30](#_Toc191670379)

[Supplementary Table 1. The search strategy in PubMed. 31](#_Toc191670380)

[Supplementary Table 2. Definitions of the MACE and repeat revascularization. 33](#_Toc191670381)

[Supplementary Table 3. Baseline characteristics. 35](#_Toc191670382)

[Supplementary Table 4. Assessment of between-study heterogeneity. 38](#_Toc191670383)

[Supplementary Table 5. Network meta-analysis for all-cause mortality in decision-making or optimization cohorts. 40](#_Toc191670384)

[Supplementary Table 6. Network meta-analysis for cardiac mortality in decision-making or optimization cohorts. 41](#_Toc191670385)

[Supplementary Table 7. Network meta-analysis for MI in decision-making or optimization cohorts. 42](#_Toc191670386)

[Supplementary Table 8. Network meta-analysis for repeat revascularization in decision-making or optimization cohorts. 43](#_Toc191670387)

[Supplementary Table 9. Network meta-analysis for stent thrombosis in decision-making or optimization cohorts. 44](#_Toc191670388)

Supplementary Figure 1. Flow diagram of the network meta-analysis.





Supplementary Figure 2. Risk of bias assessment in the randomized parallel-arm studies.


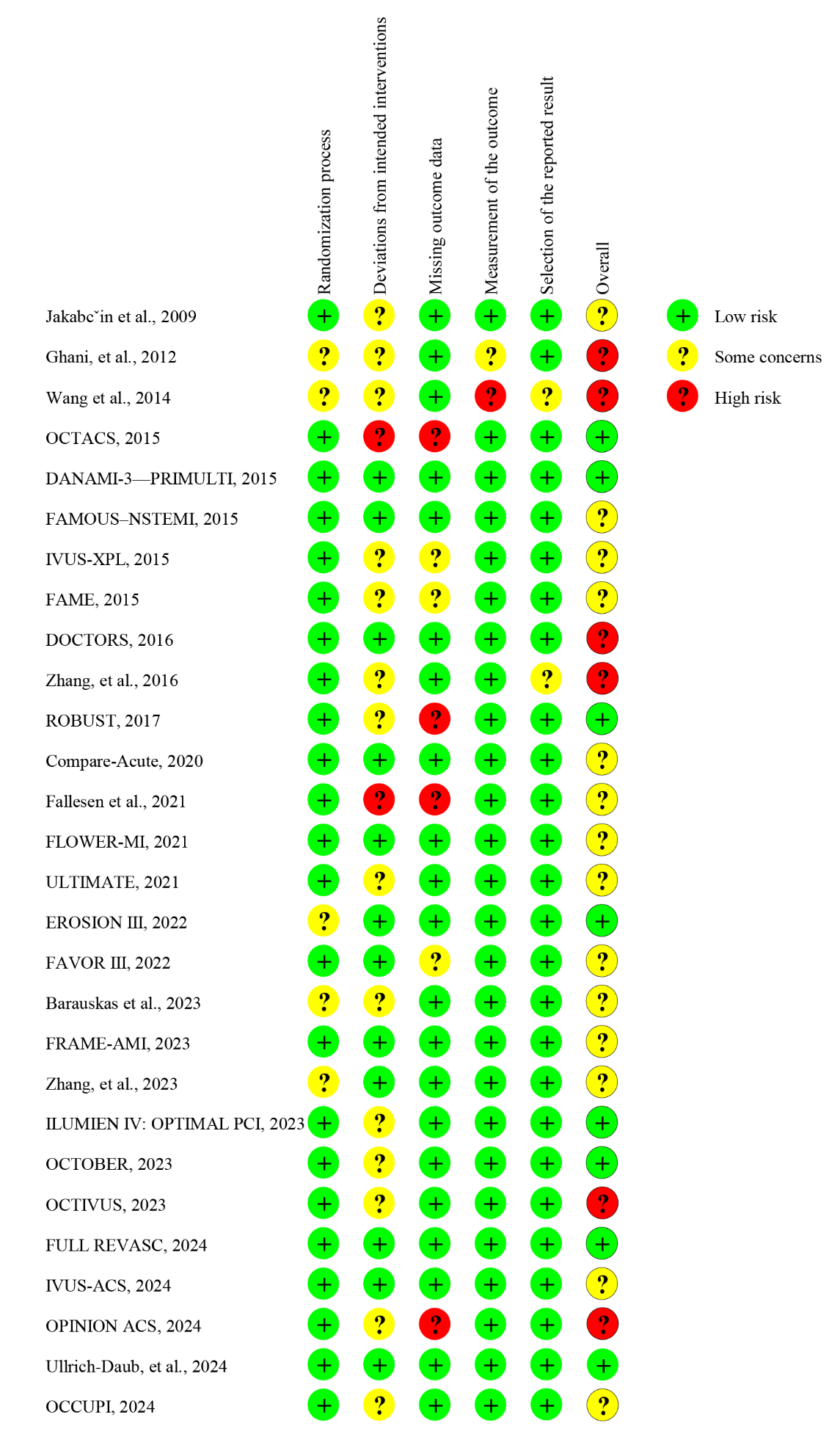


Supplementary Figure 3. Comparison-adjusted funnel plots for MACE.


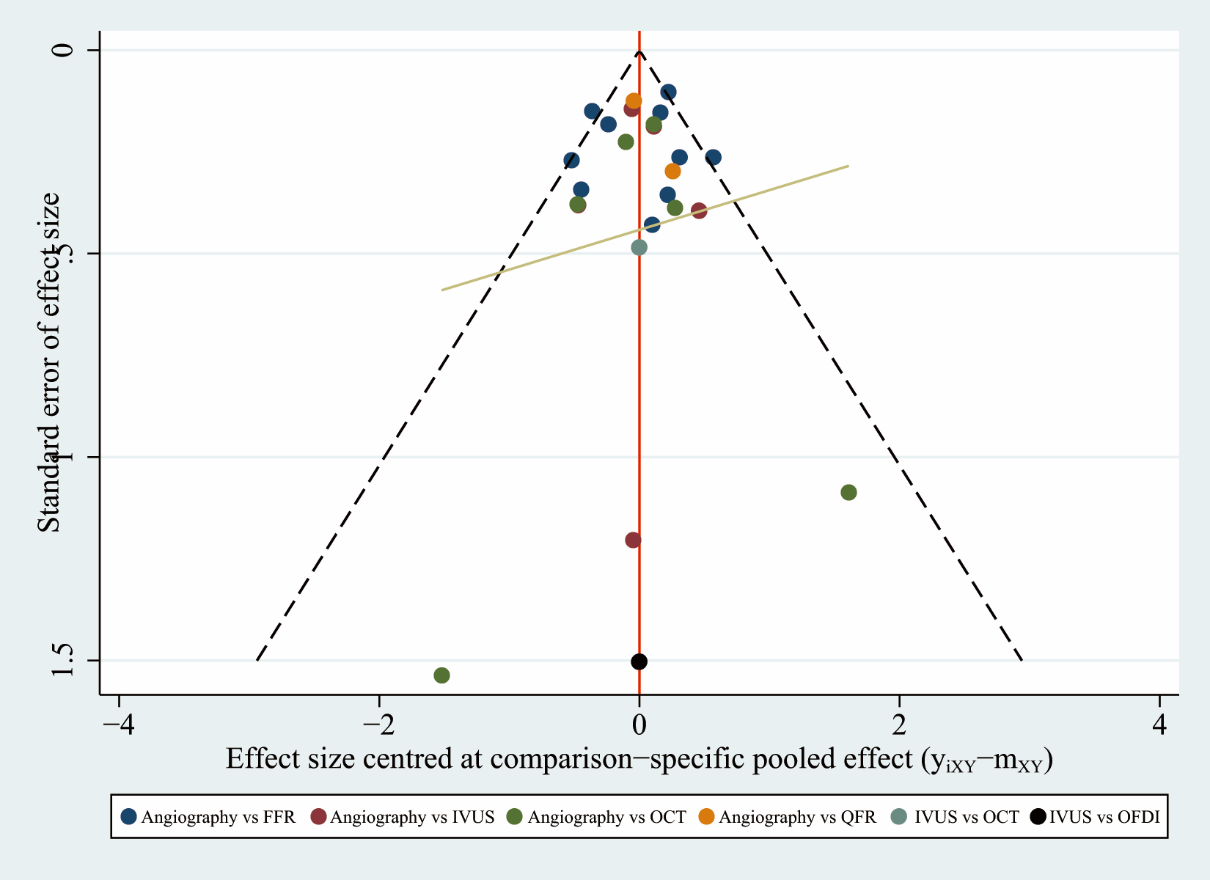


Supplementary Figure 4. Comparison-adjusted funnel plots for all-cause mortality.


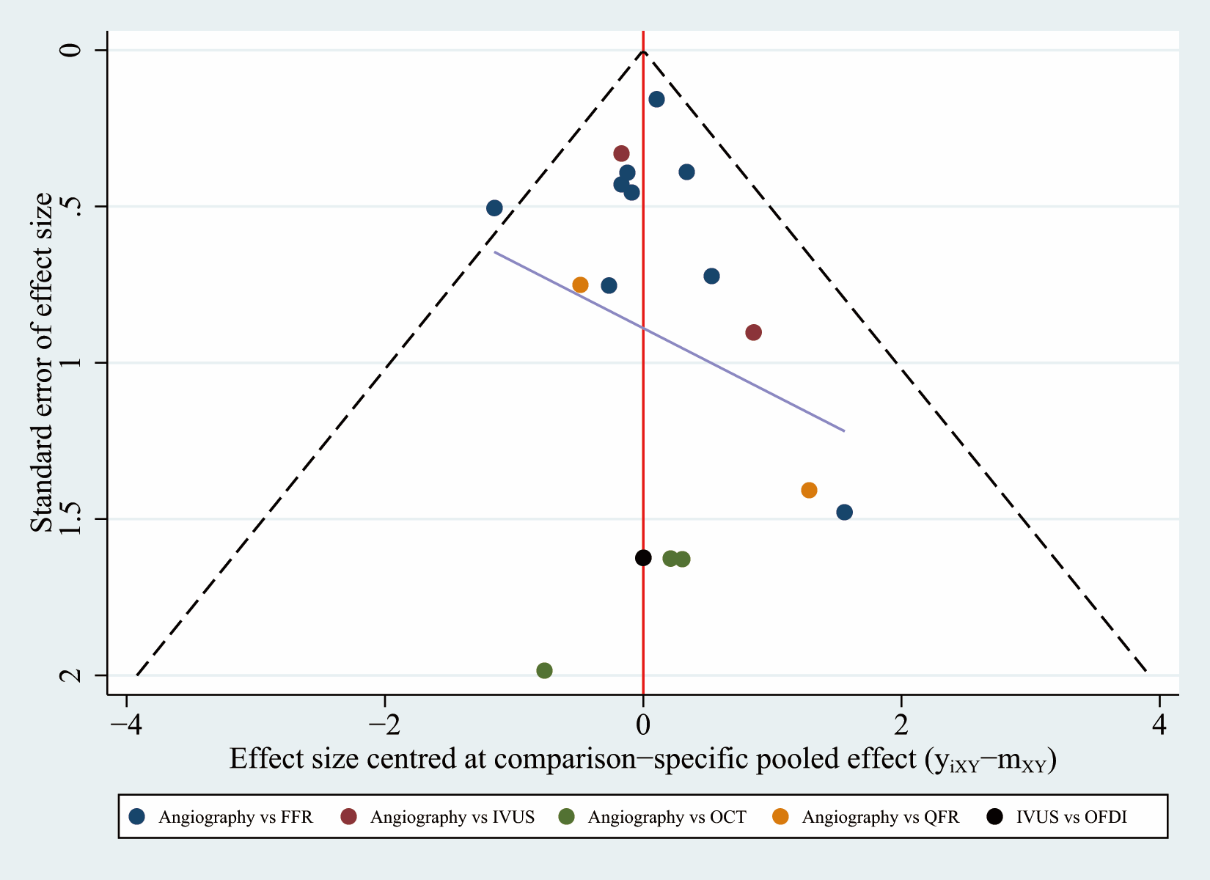


Supplementary Figure 5. Comparison-adjusted funnel plots for cardiac mortality.
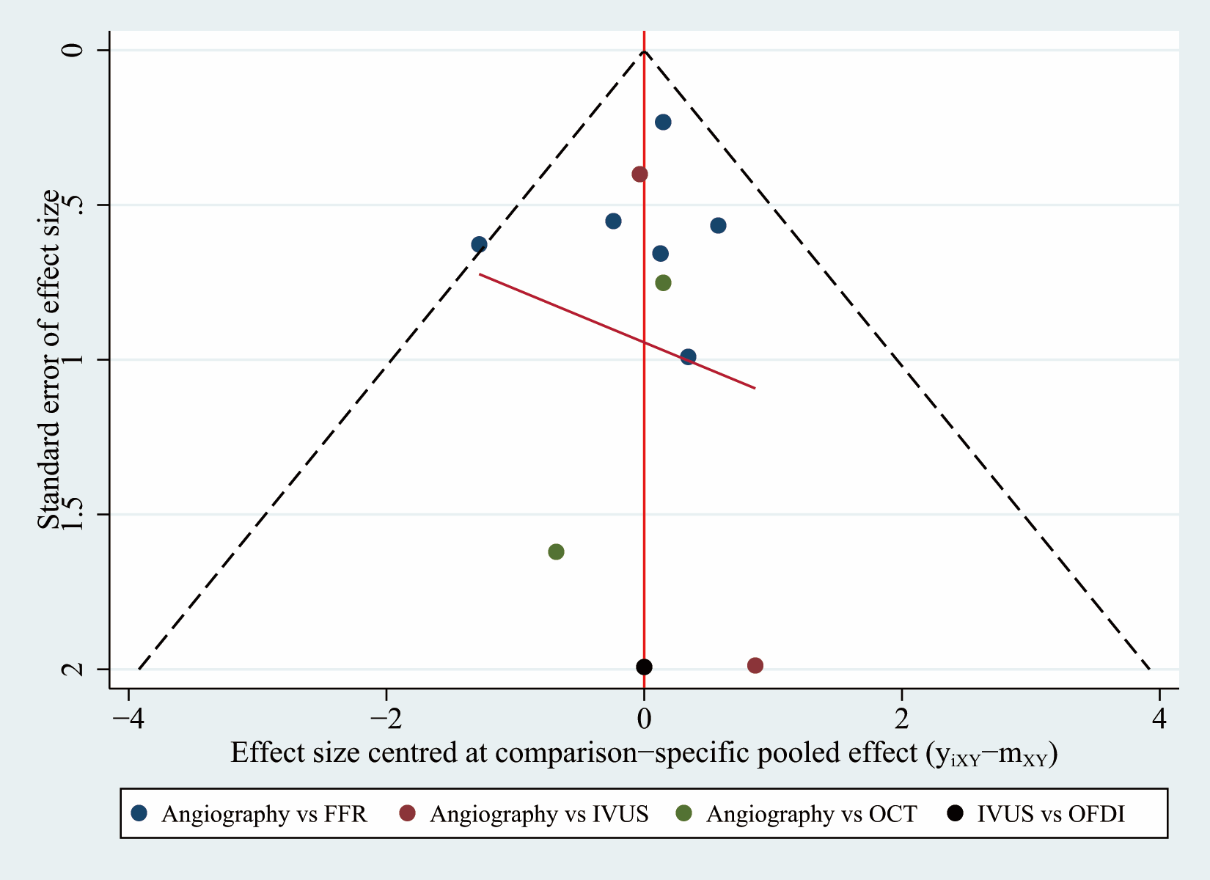


Supplementary Figure 6. Comparison-adjusted funnel plots for MI.


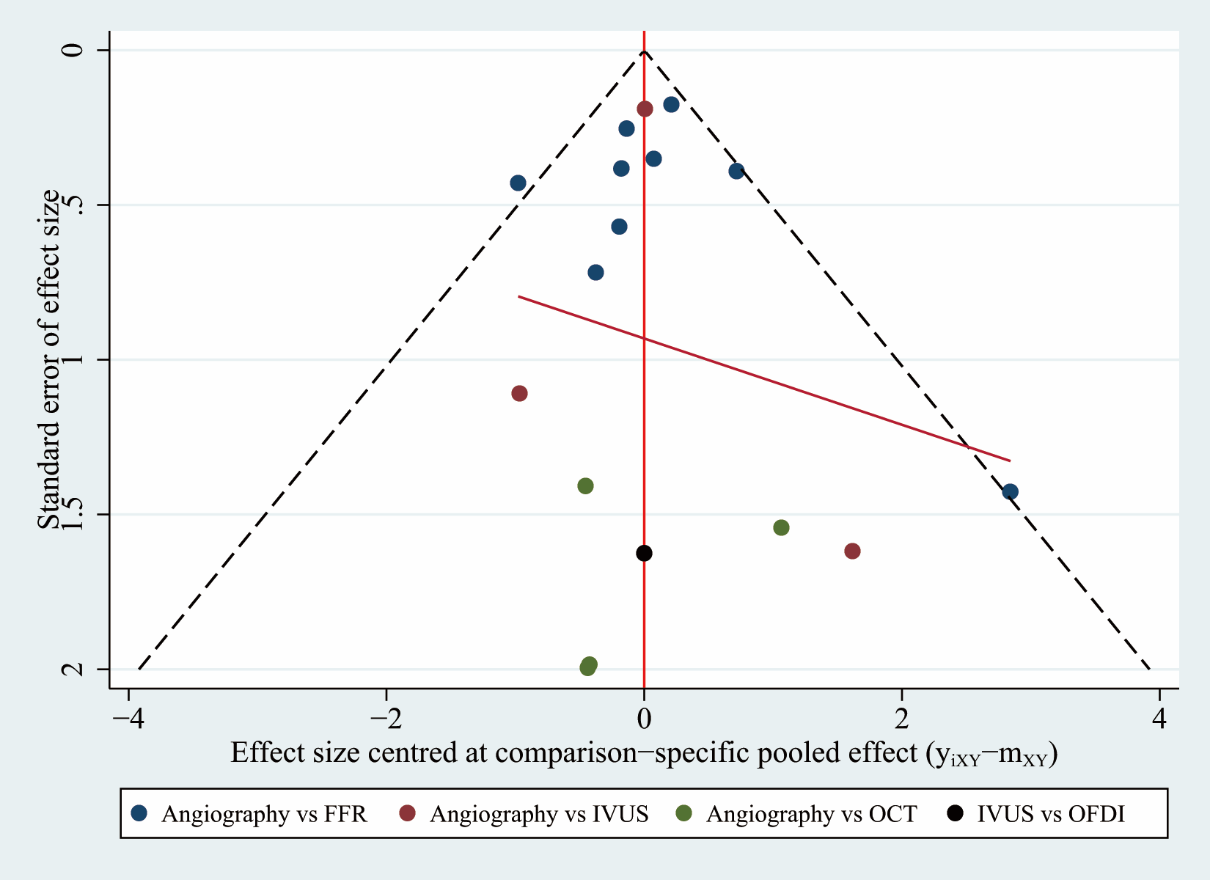


Supplementary Figure 7. Comparison-adjusted funnel plots for repeat revascularization.
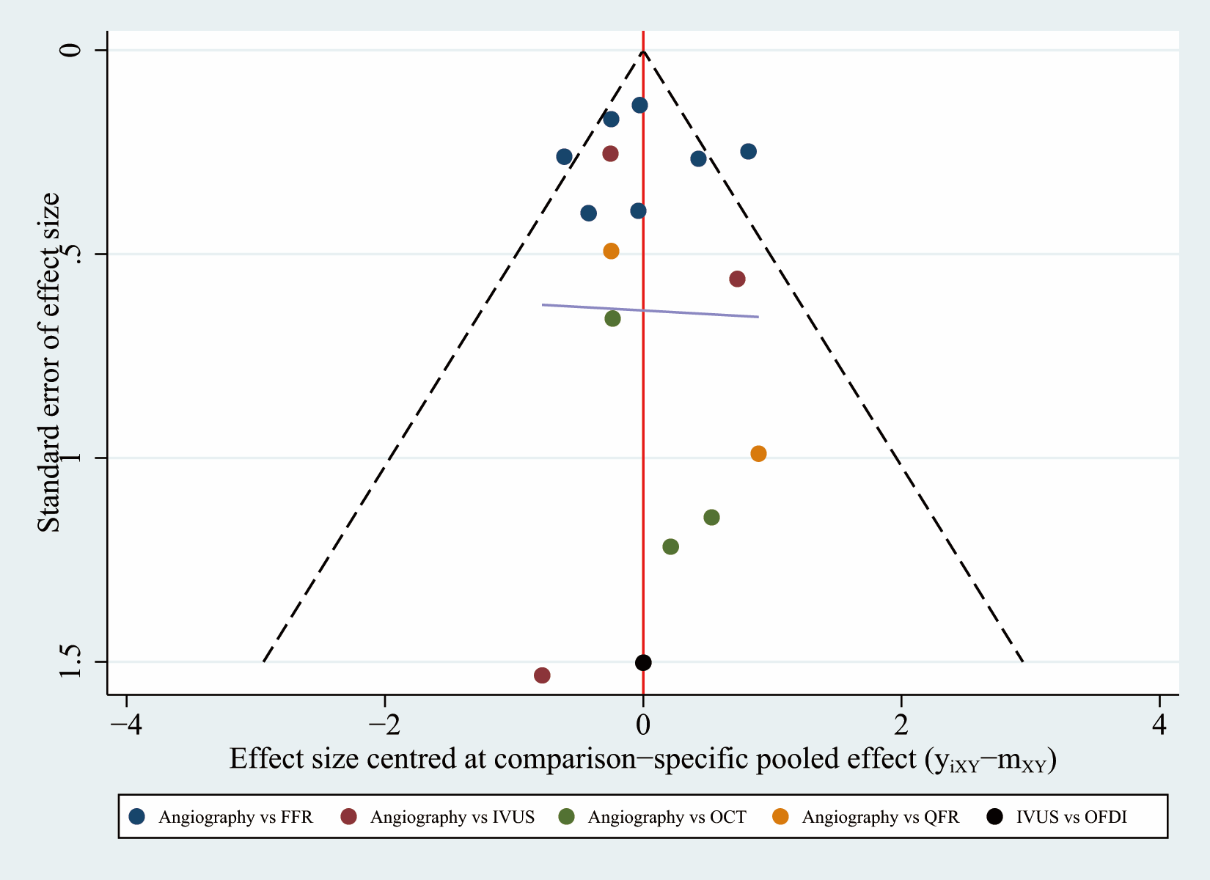


Supplementary Figure 8. Comparison-adjusted funnel plots for stent thrombosis.


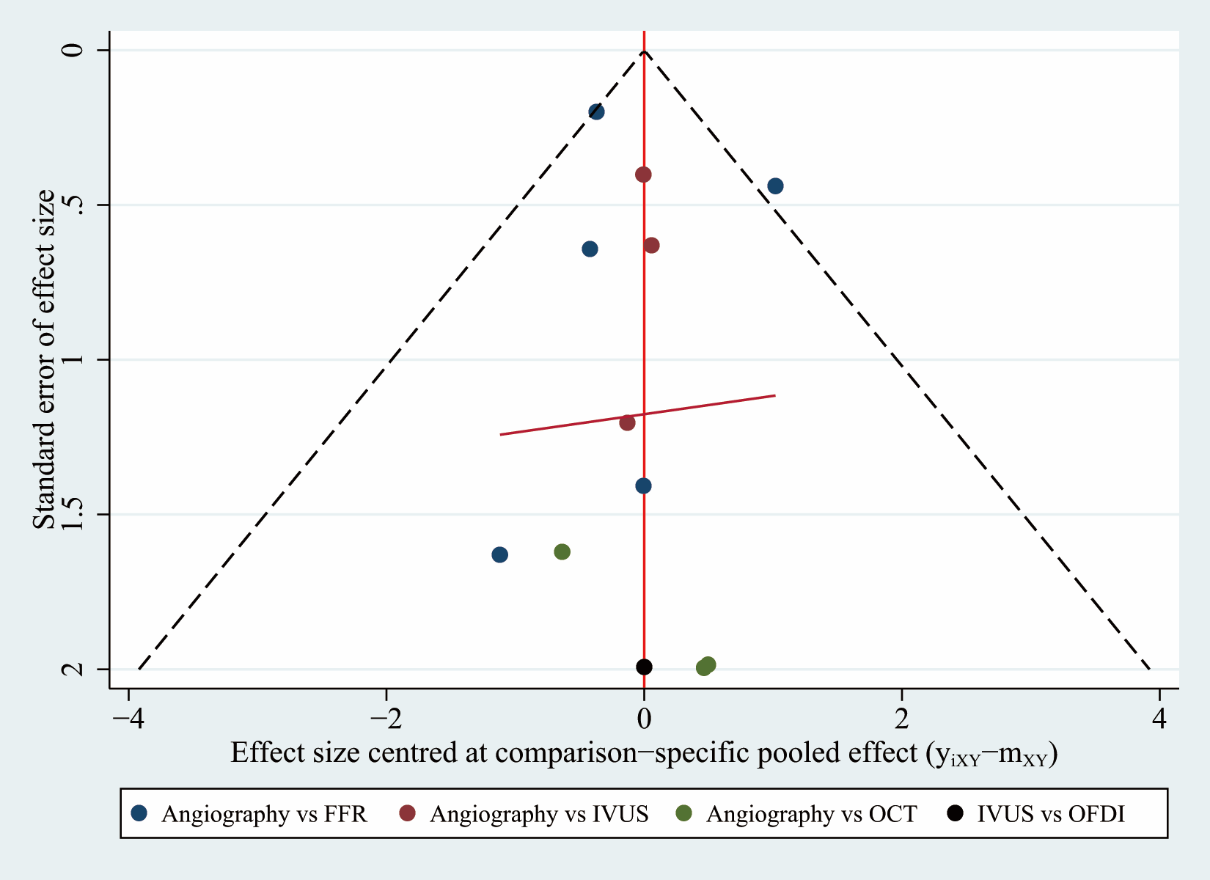


Supplementary Figure 9. Network plot of intravascular imaging-guided, physiology-guided, and angiography-guided PCI for all-cause mortality.


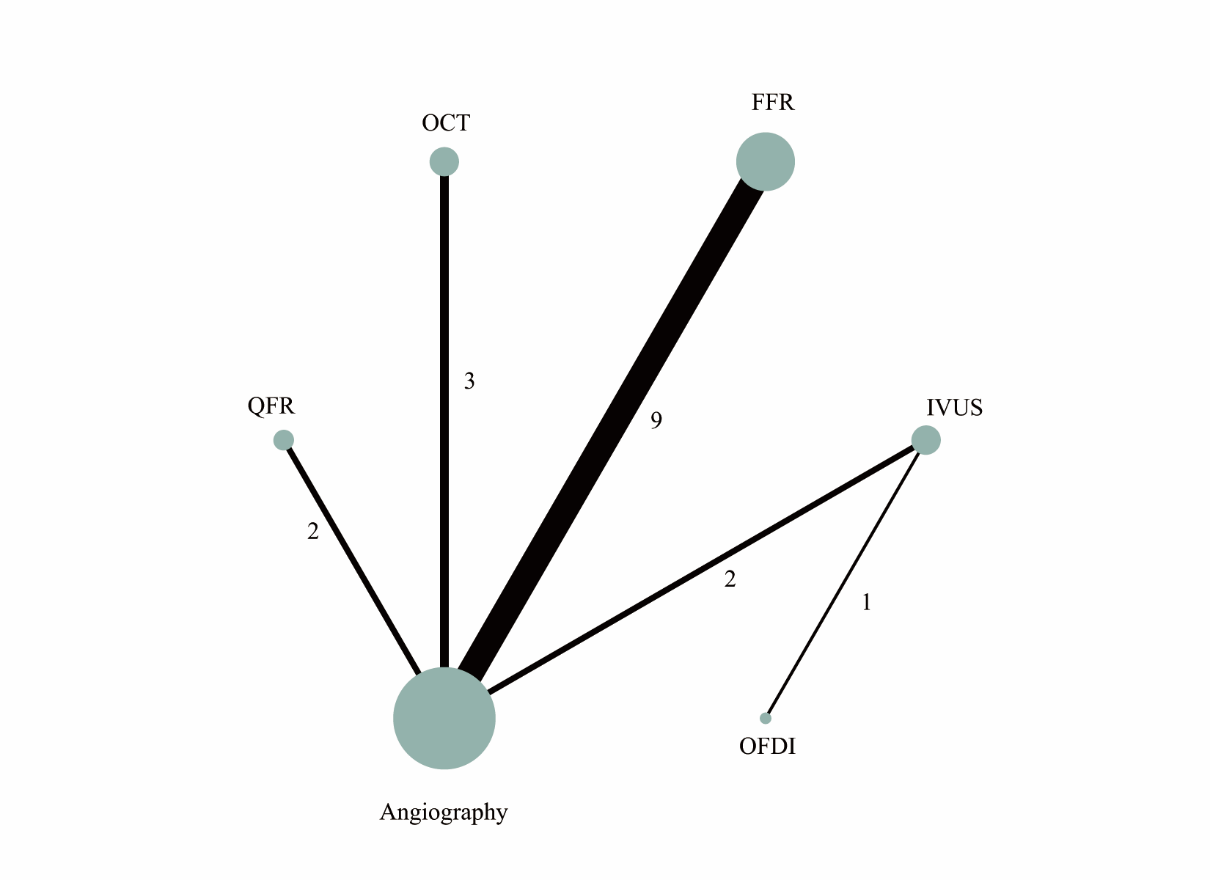


Supplementary Figure 10. Network plot of intravascular imaging-guided, physiology-guided, and angiography-guided PCI for cardiac mortality.


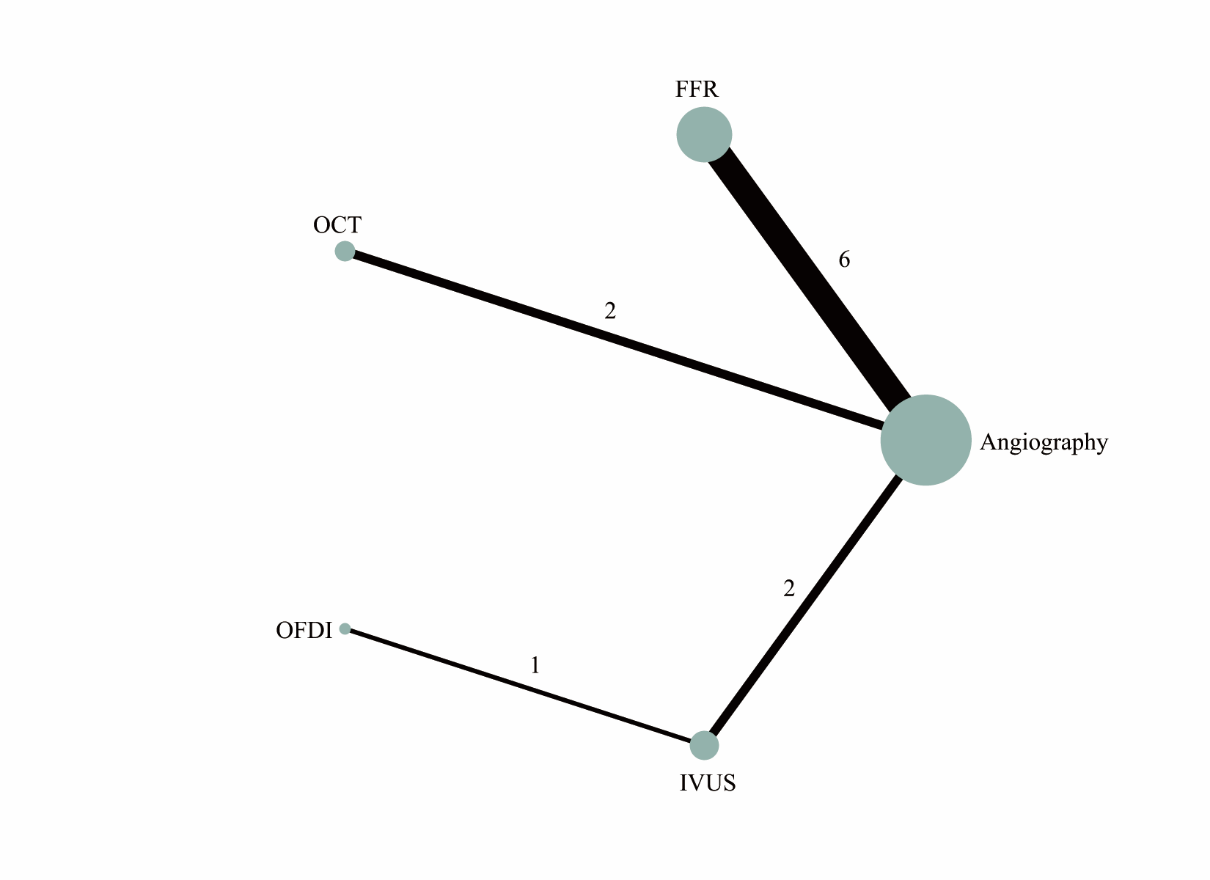


Supplementary Figure 11. Network plot of intravascular imaging-guided, physiology-guided, and angiography-guided PCI for MI.


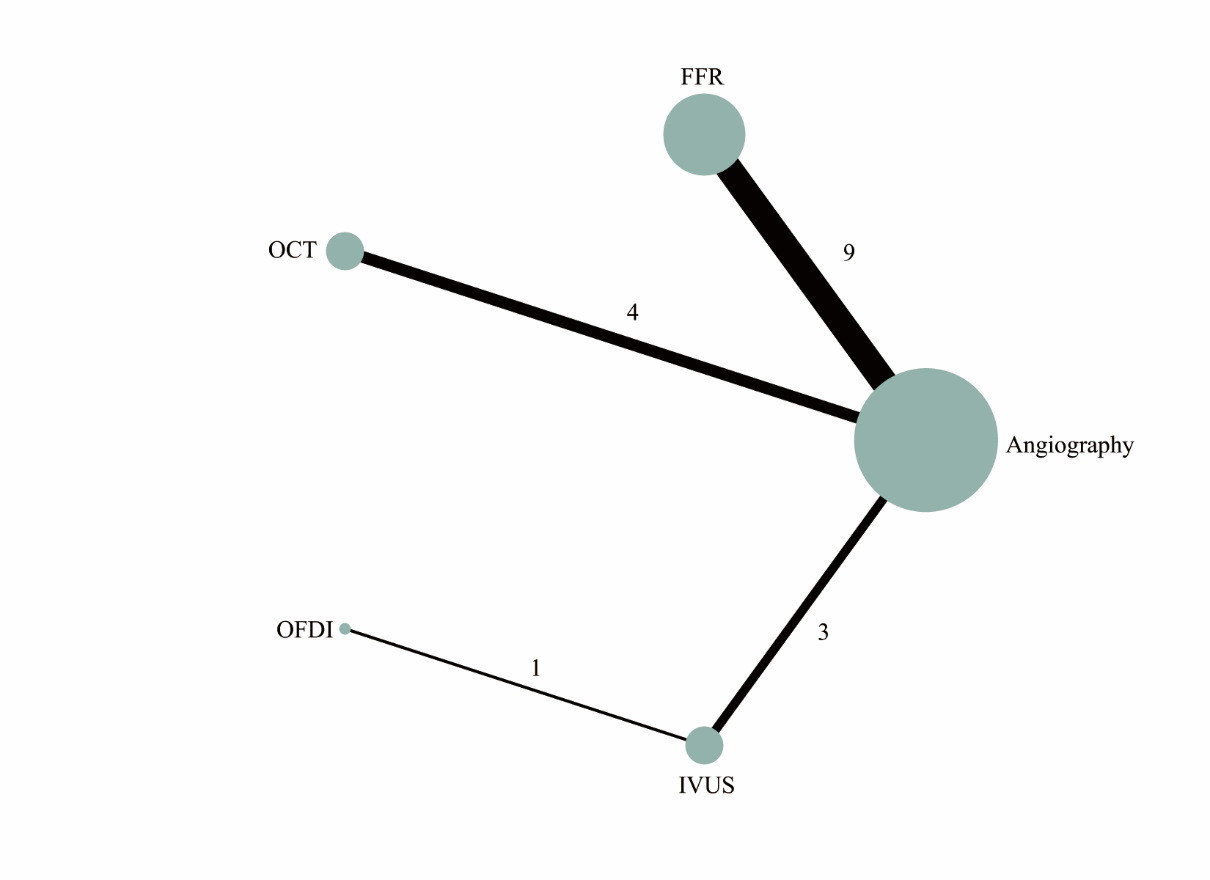


Supplementary Figure 12. Network plot of intravascular imaging-guided, physiology-guided, and angiography-guided PCI for repeat revascularization.


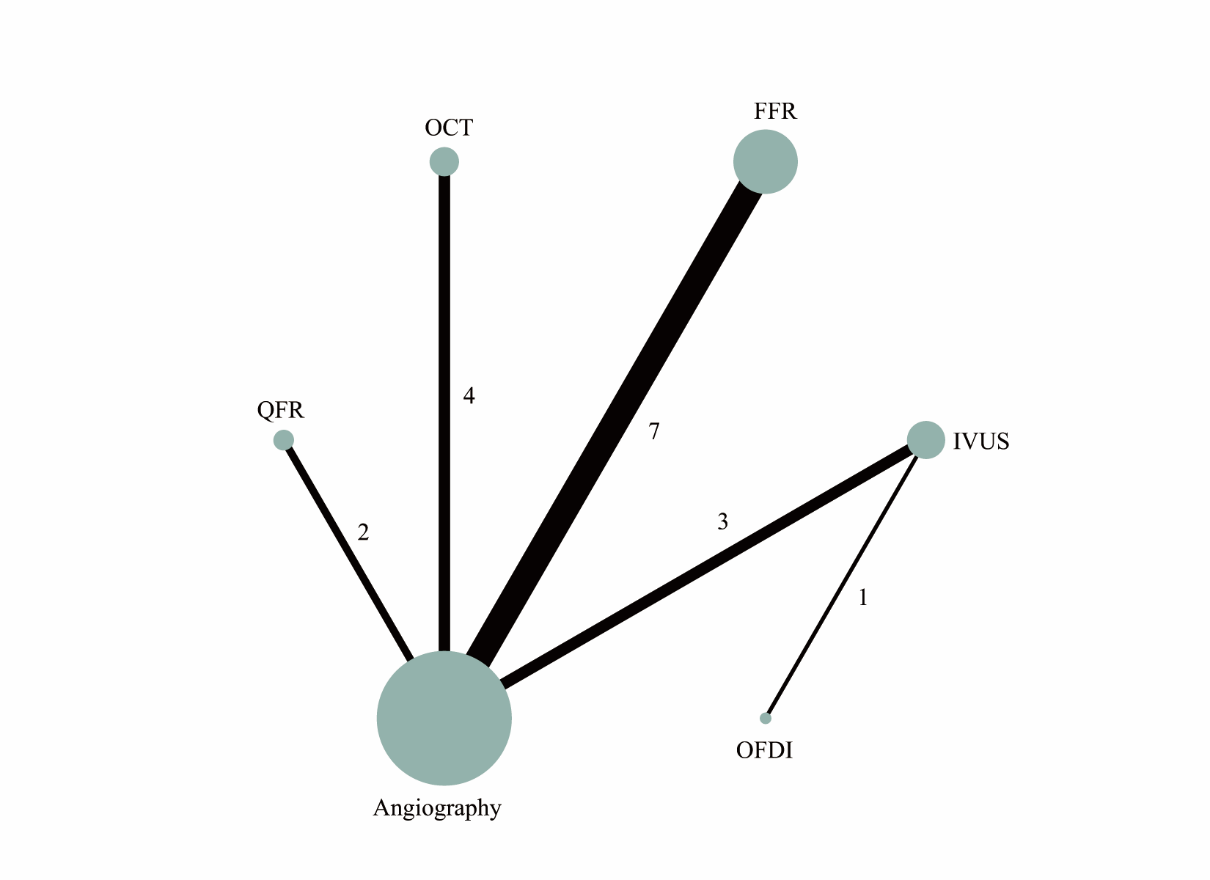


Supplementary Figure 13. Network plot of intravascular imaging-guided, physiology-guided, and angiography-guided PCI for stent thrombosis.


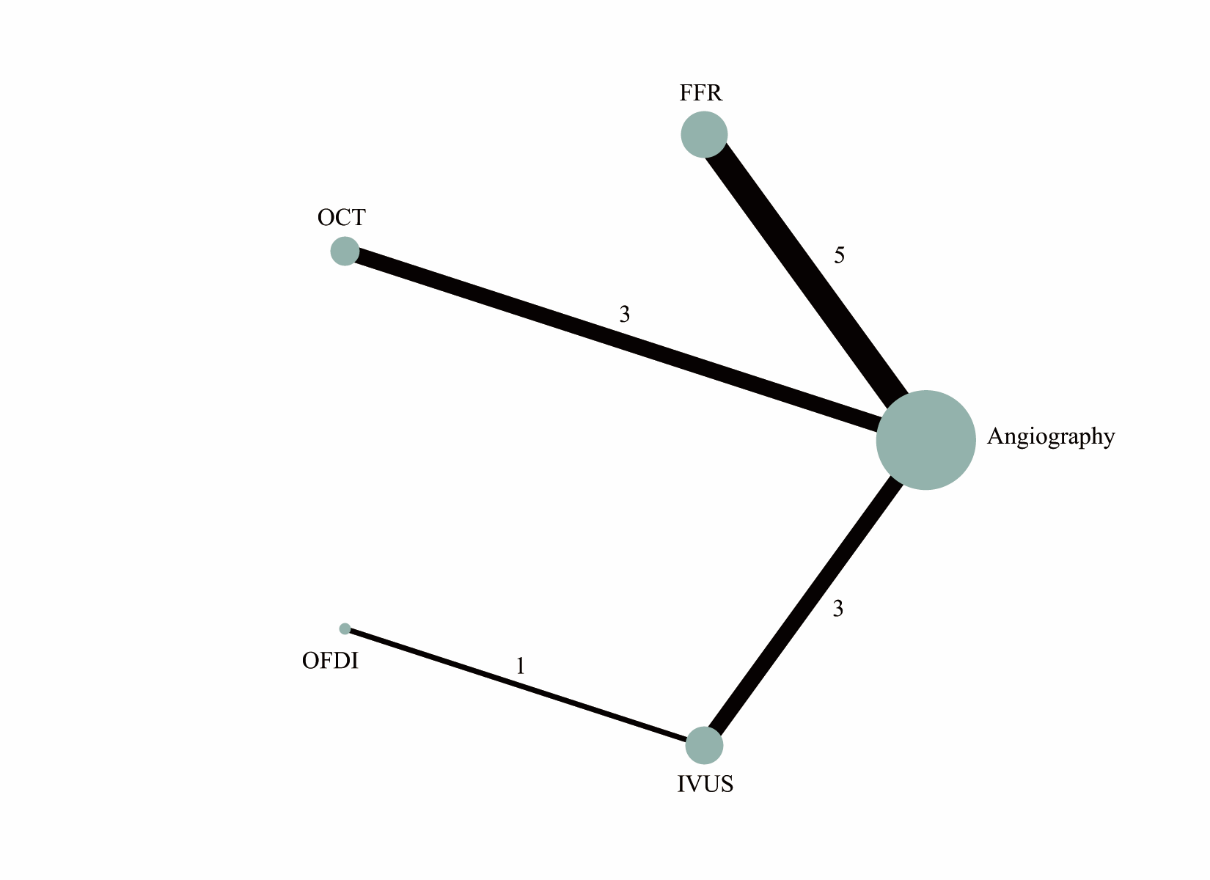


Supplementary Figure 14. Network meta-analysis for all-cause mortality.


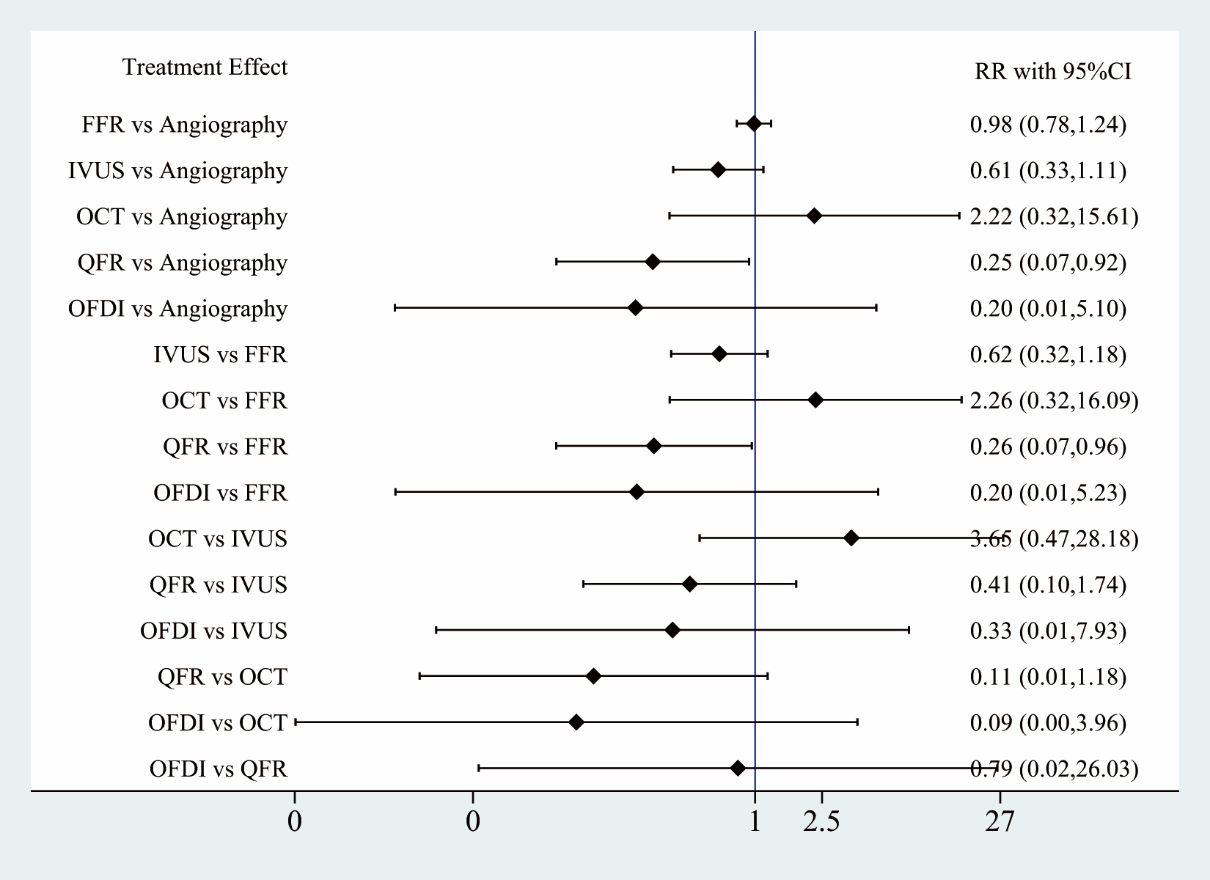


Supplementary Figure 15. Network meta-analysis for cardiac mortality.


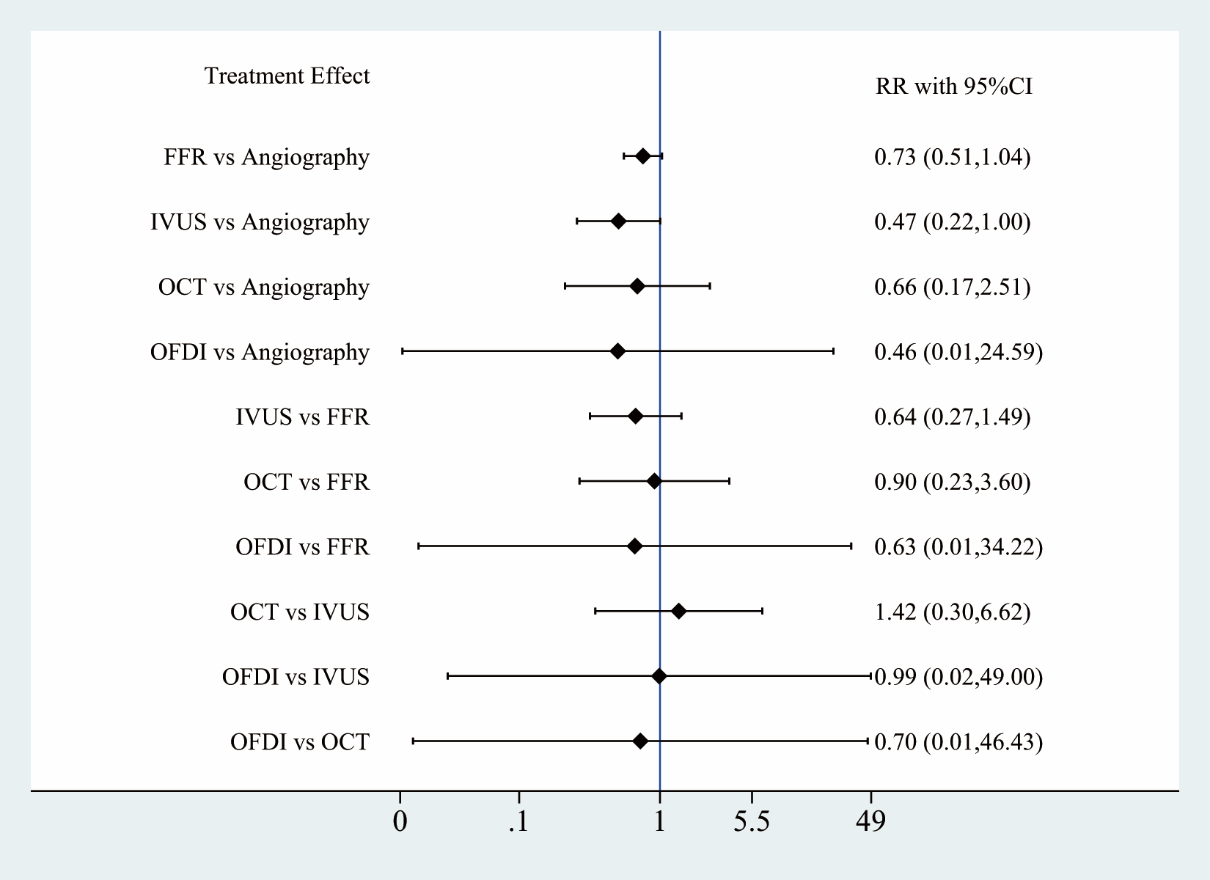


Supplementary Figure 16. Network meta-analysis for MI.


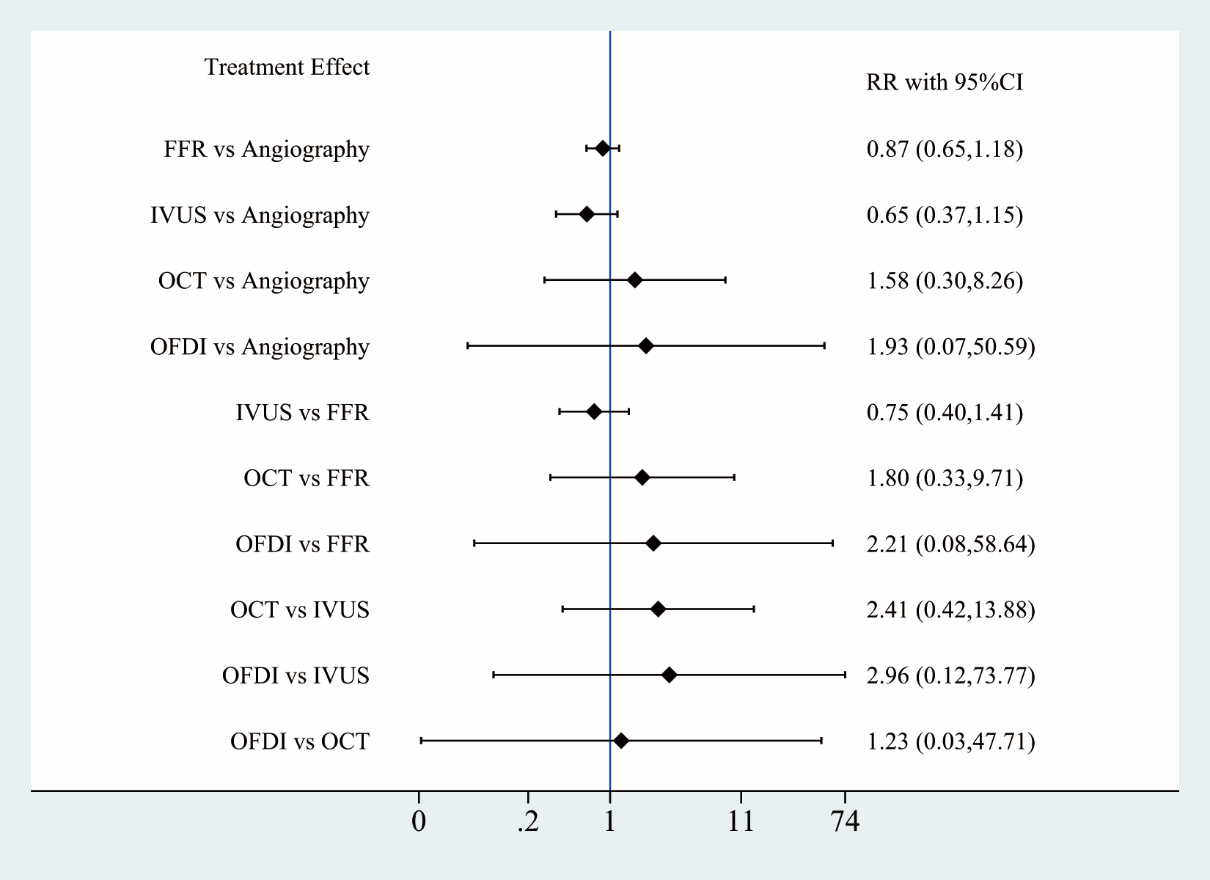


Supplementary Figure 17. Network meta-analysis for repeat revascularization.


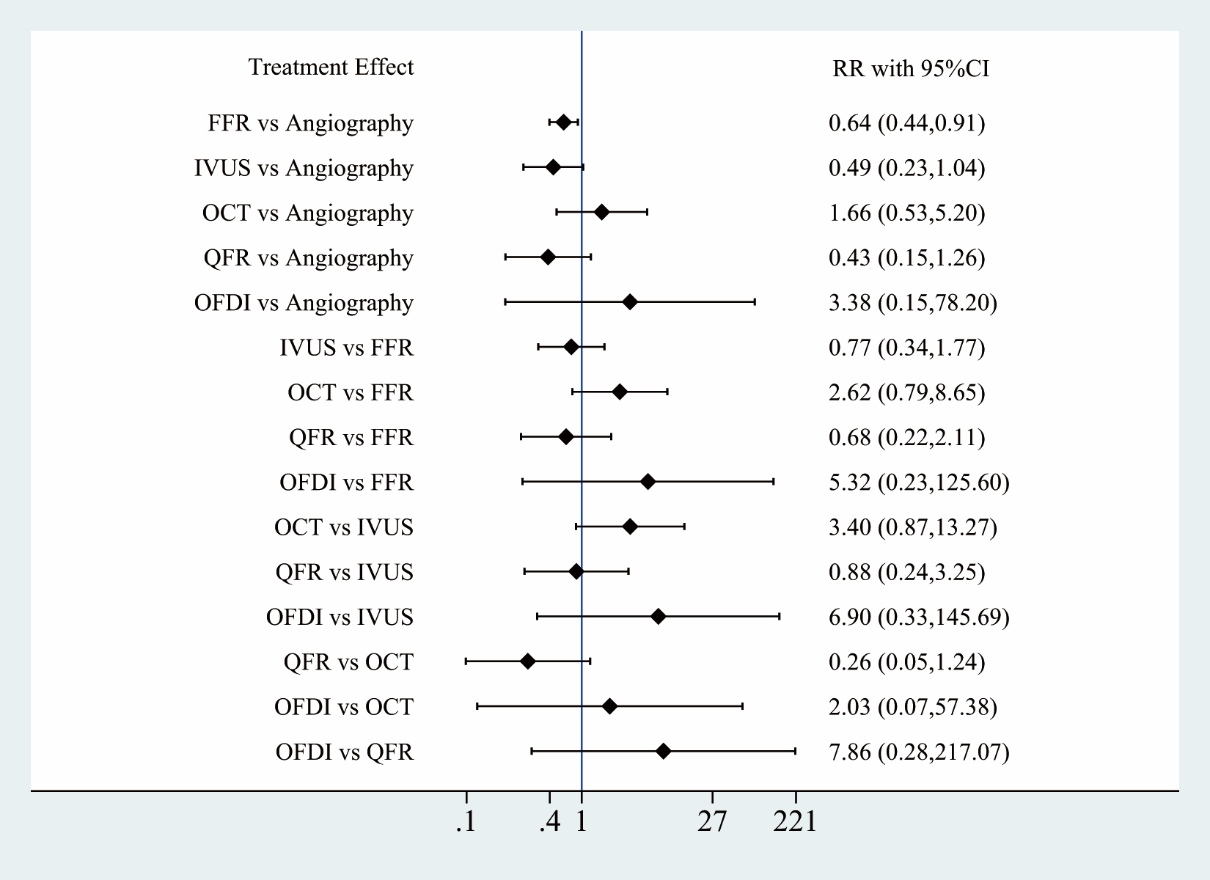


Supplementary Figure 18. Network meta-analysis for stent thrombosis.


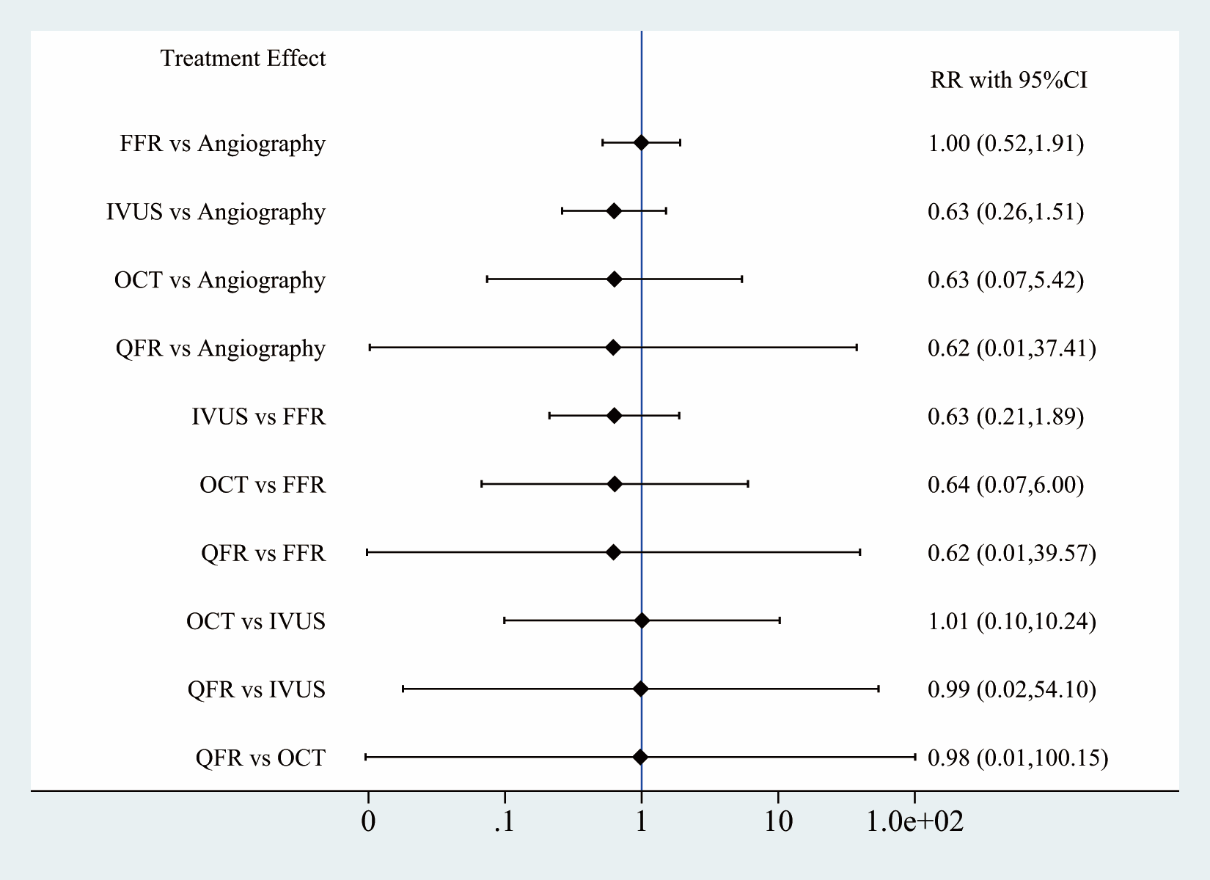


Supplementary Figure 19. Rankogram of the six strategies for all-cause mortality.


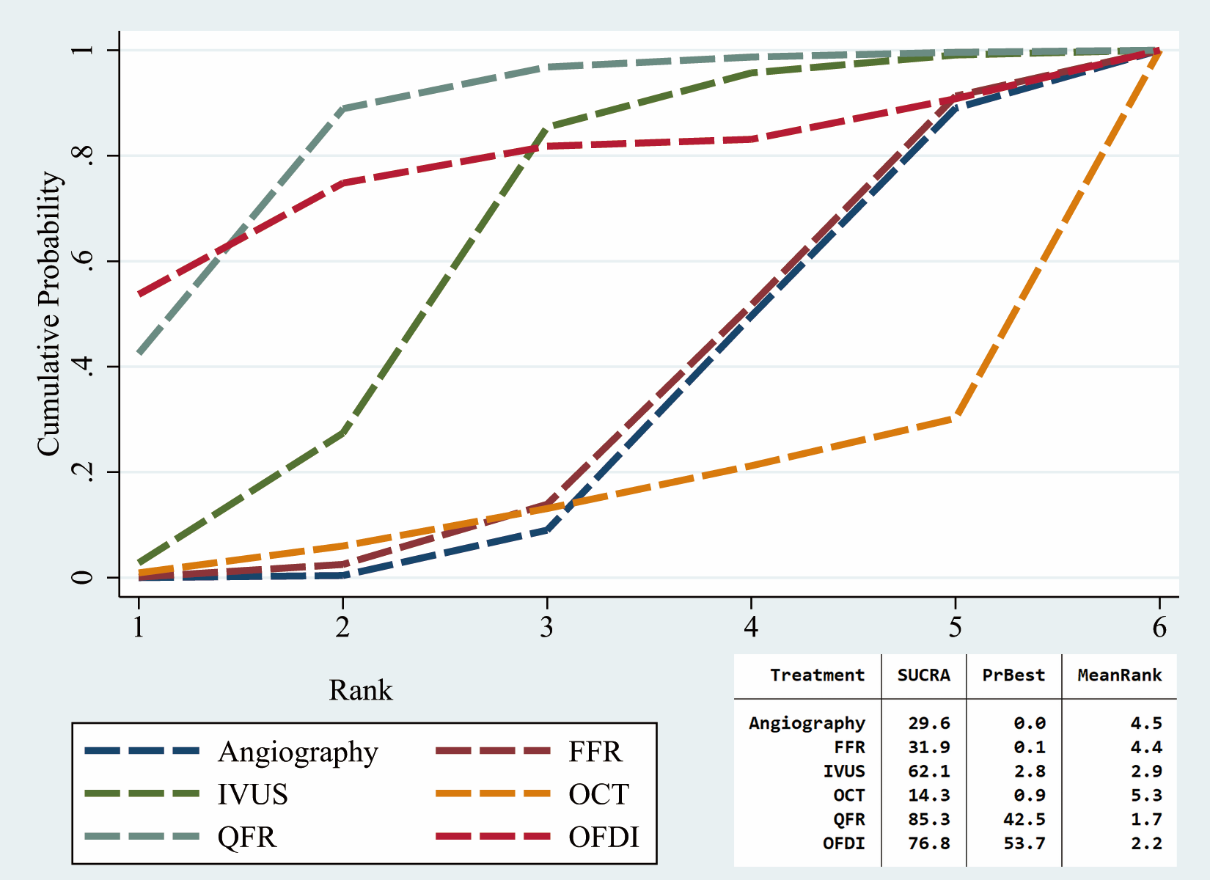


Supplementary Figure 20. Rankogram of the five strategies for cardiac mortality.


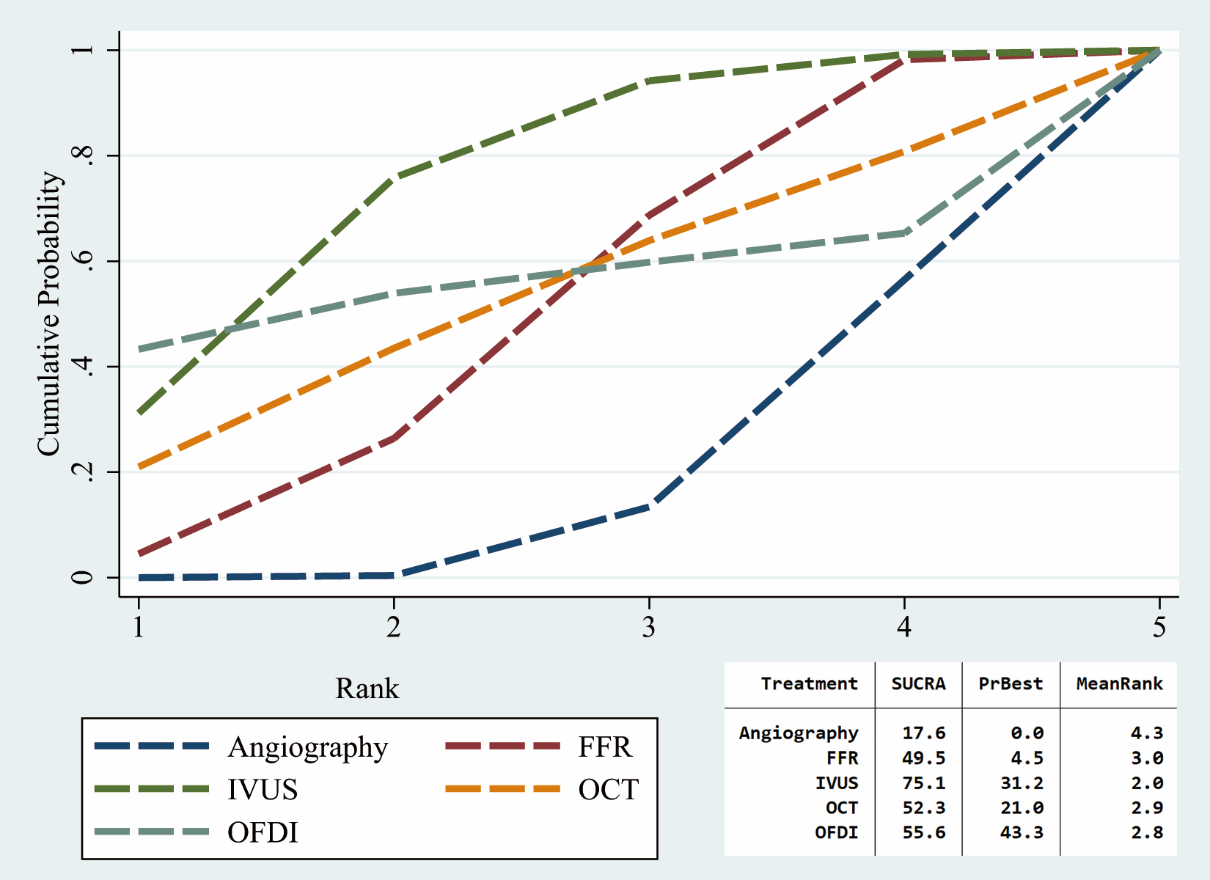


Supplementary Figure 21. Rankogram of the five strategies for MI.


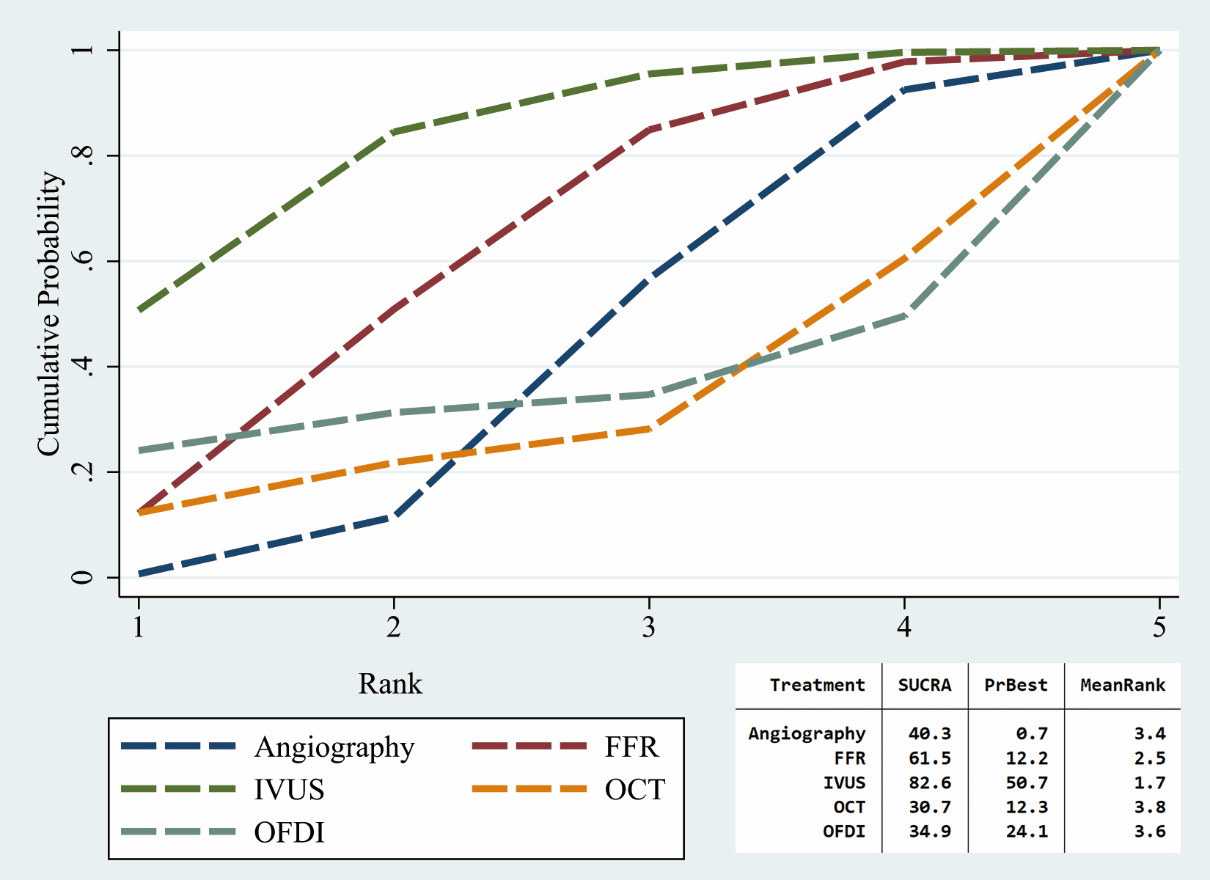


Supplementary Figure 22. Rankogram of the six strategies for repeat revascularization.


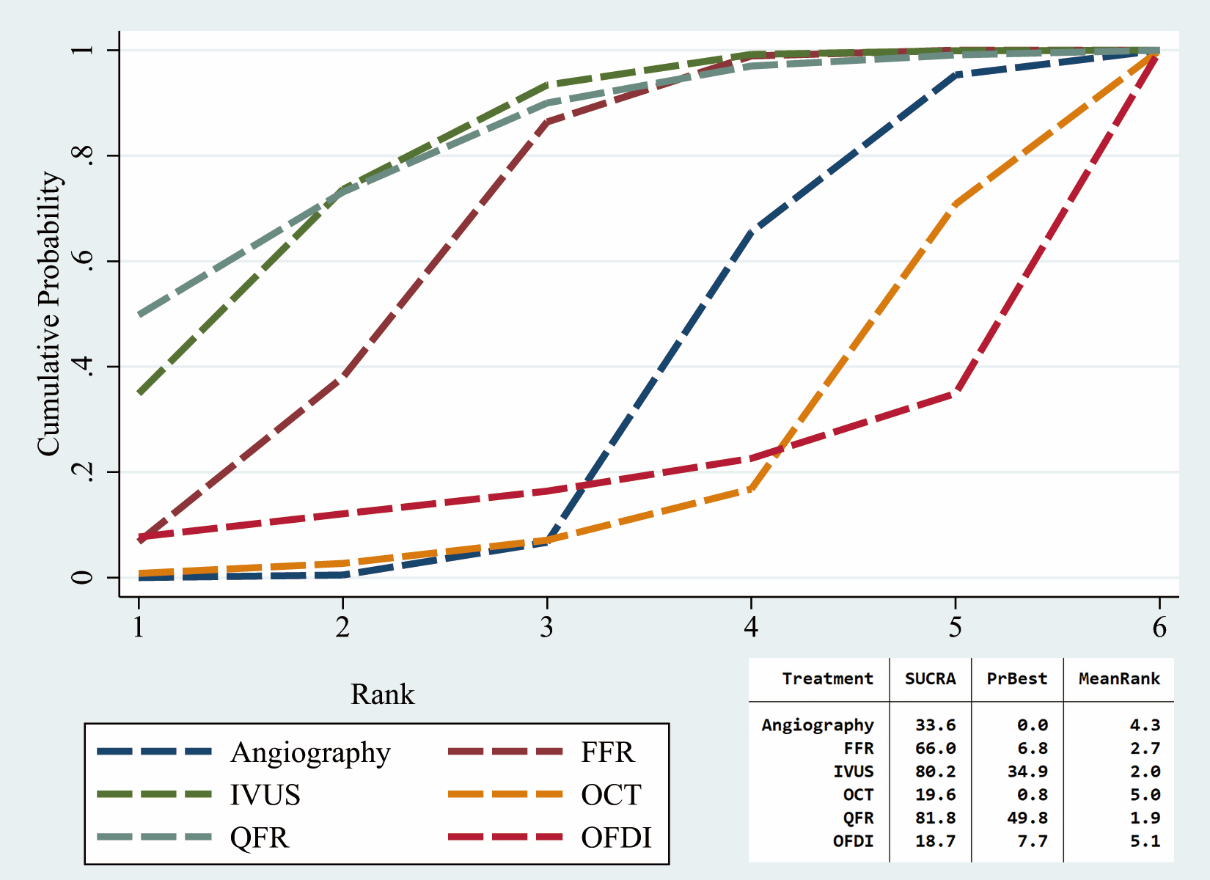


Supplementary Figure 23. Rankogram of the five strategies for stent thrombosis.


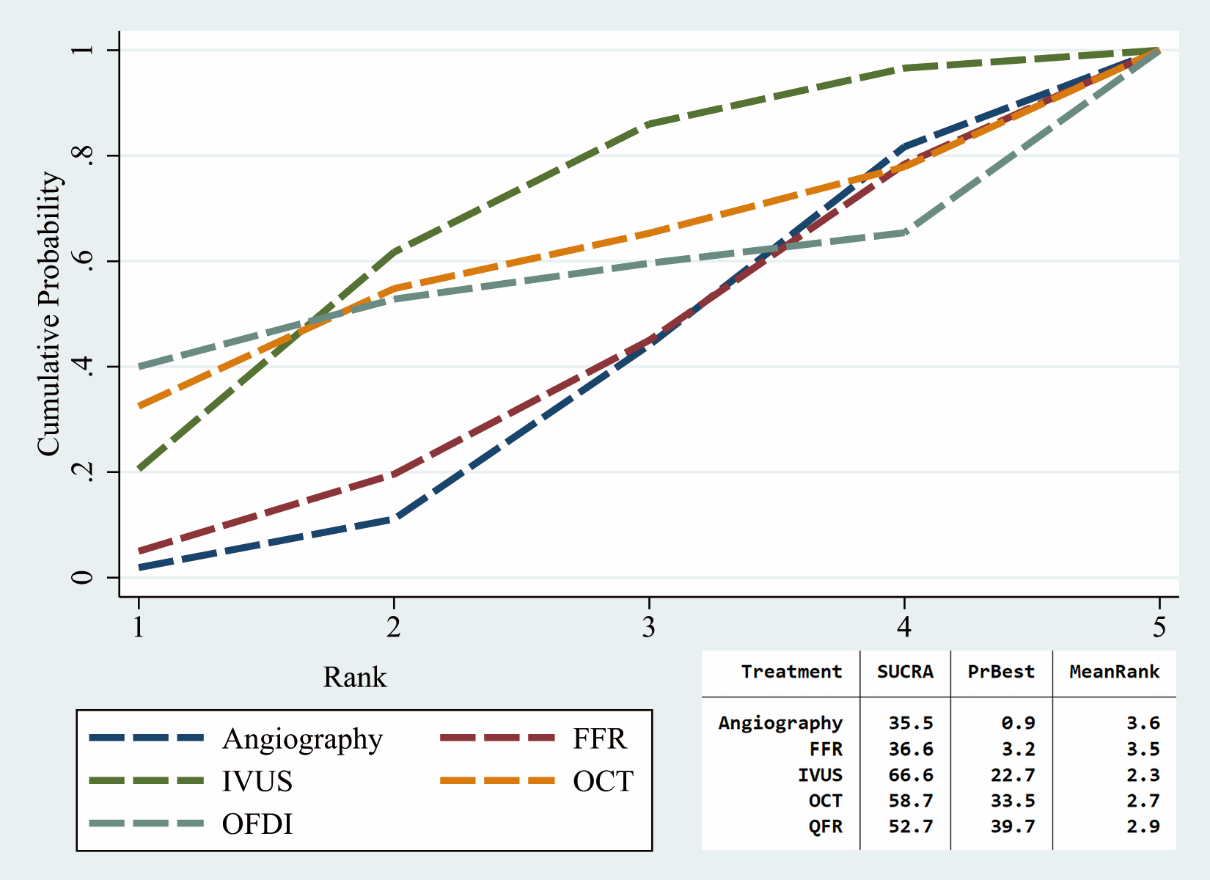


Supplementary Figure 24. Network meta-analysis for all-cause mortality in sensitivity analysis.


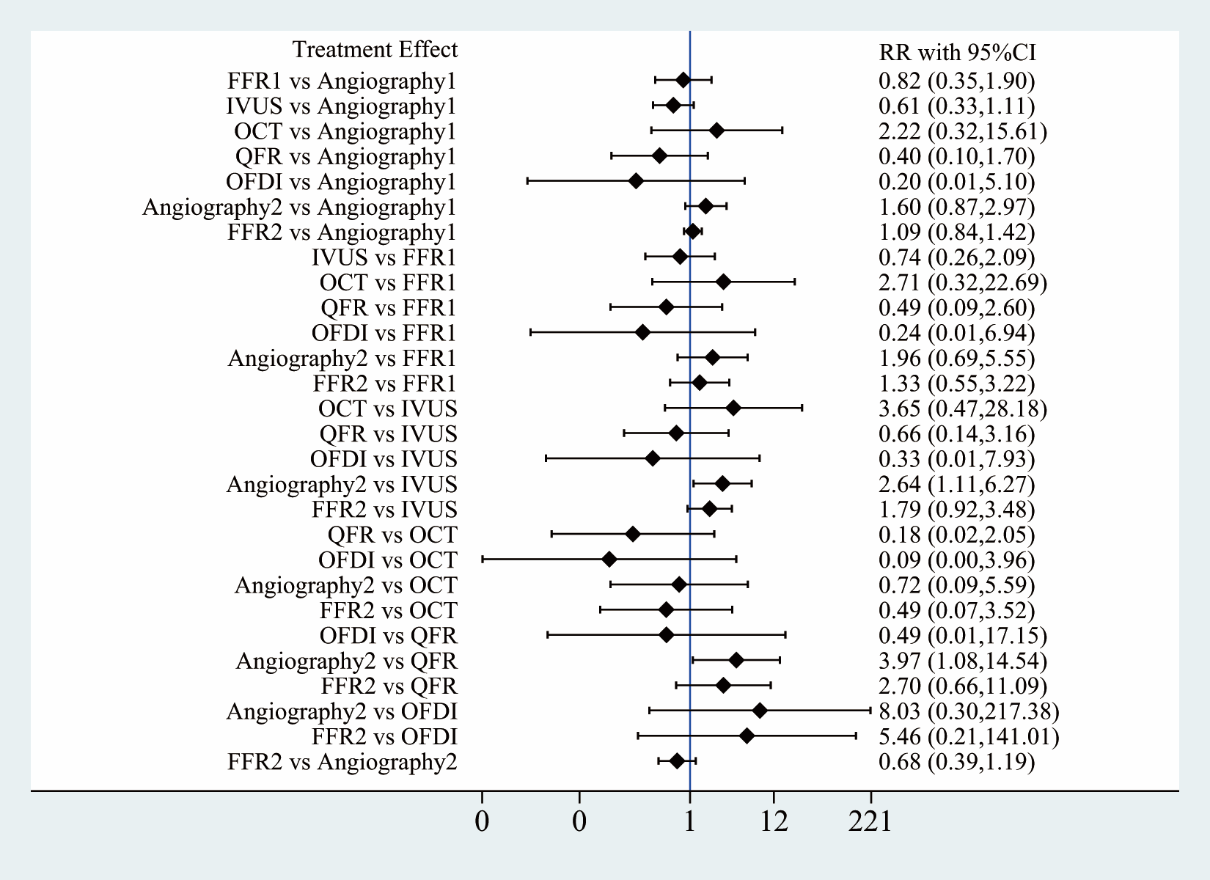


Supplementary Figure 25. Network meta-analysis for cardiac mortality in sensitivity analysis.


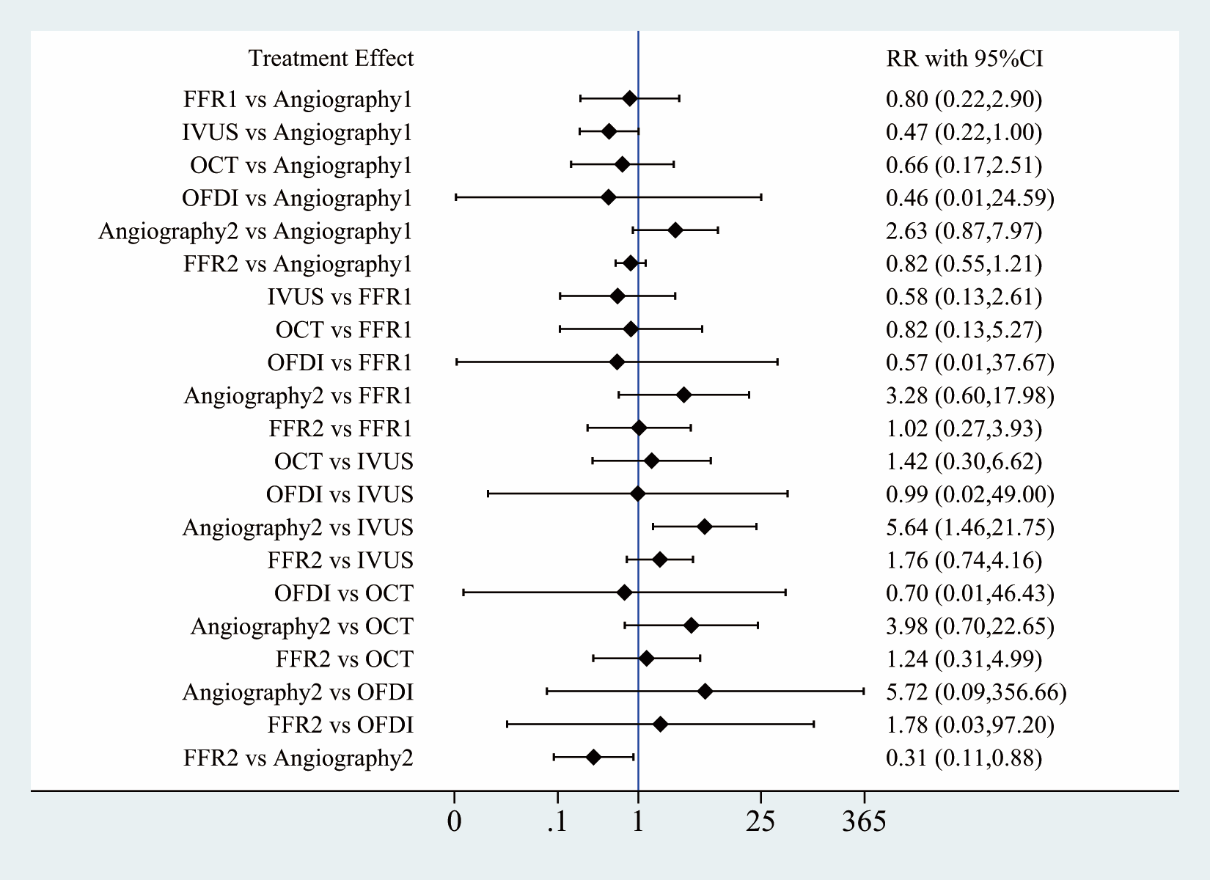


Supplementary Figure 26. Network meta-analysis for MI in sensitivity analysis.


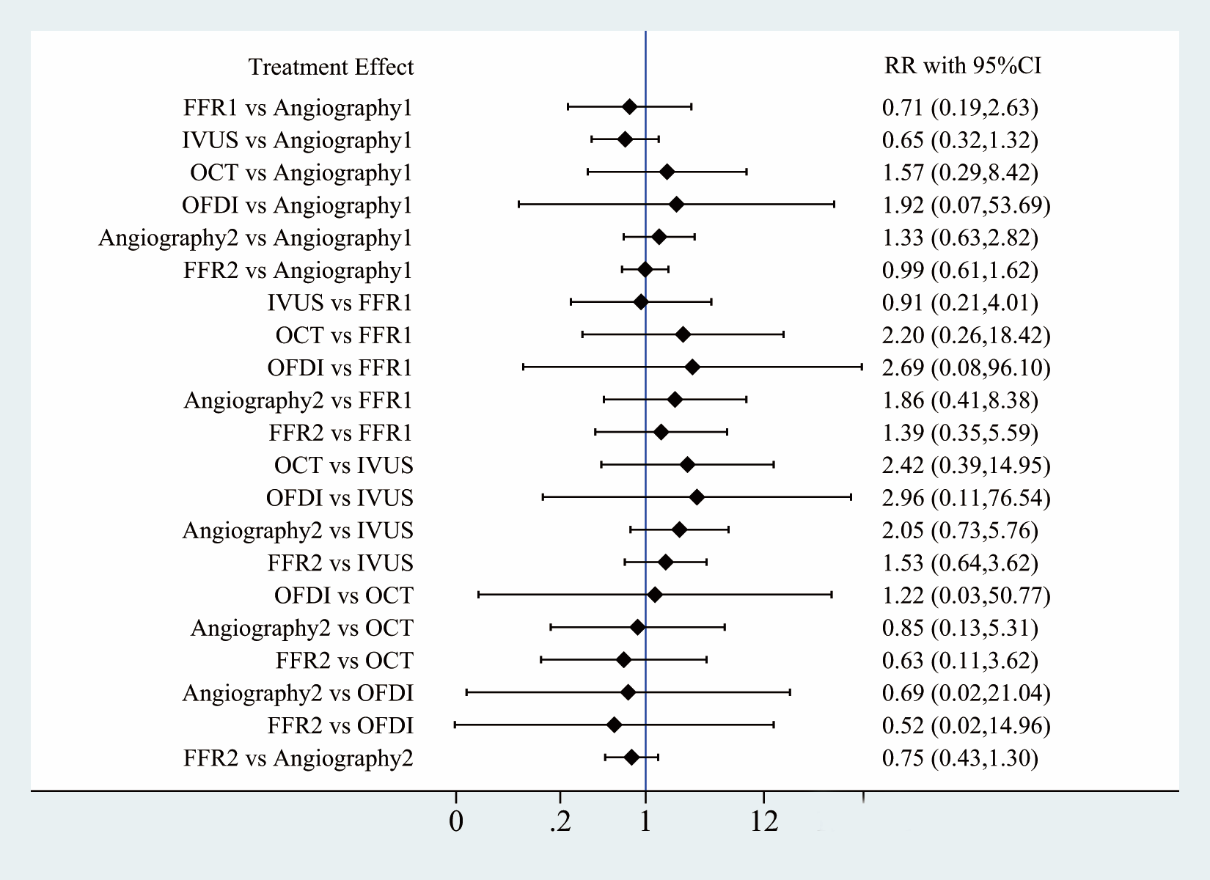


Supplementary Figure 27. Network meta-analysis for repeat revascularization in sensitivity analysis.


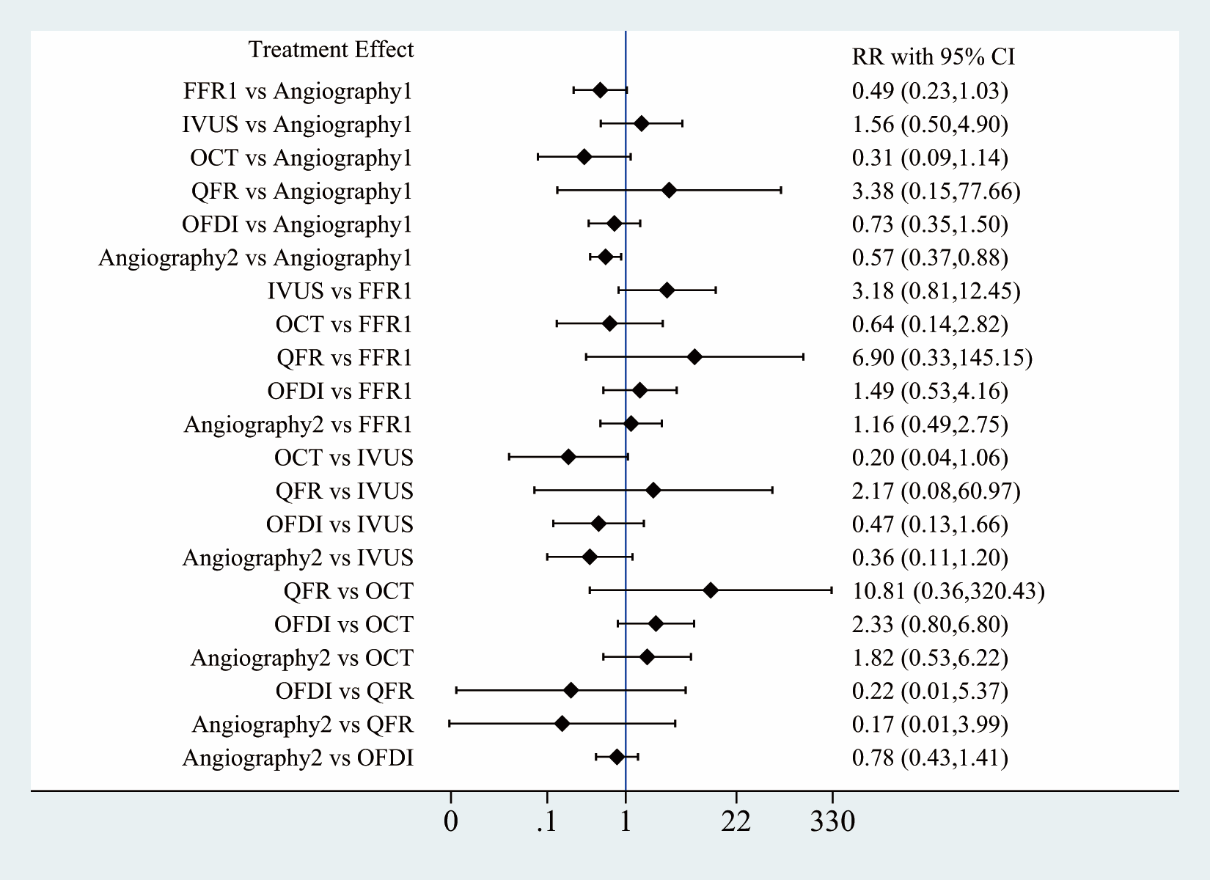


Supplementary Figure 28. Network meta-analysis for stent thrombosis in sensitivity analysis.


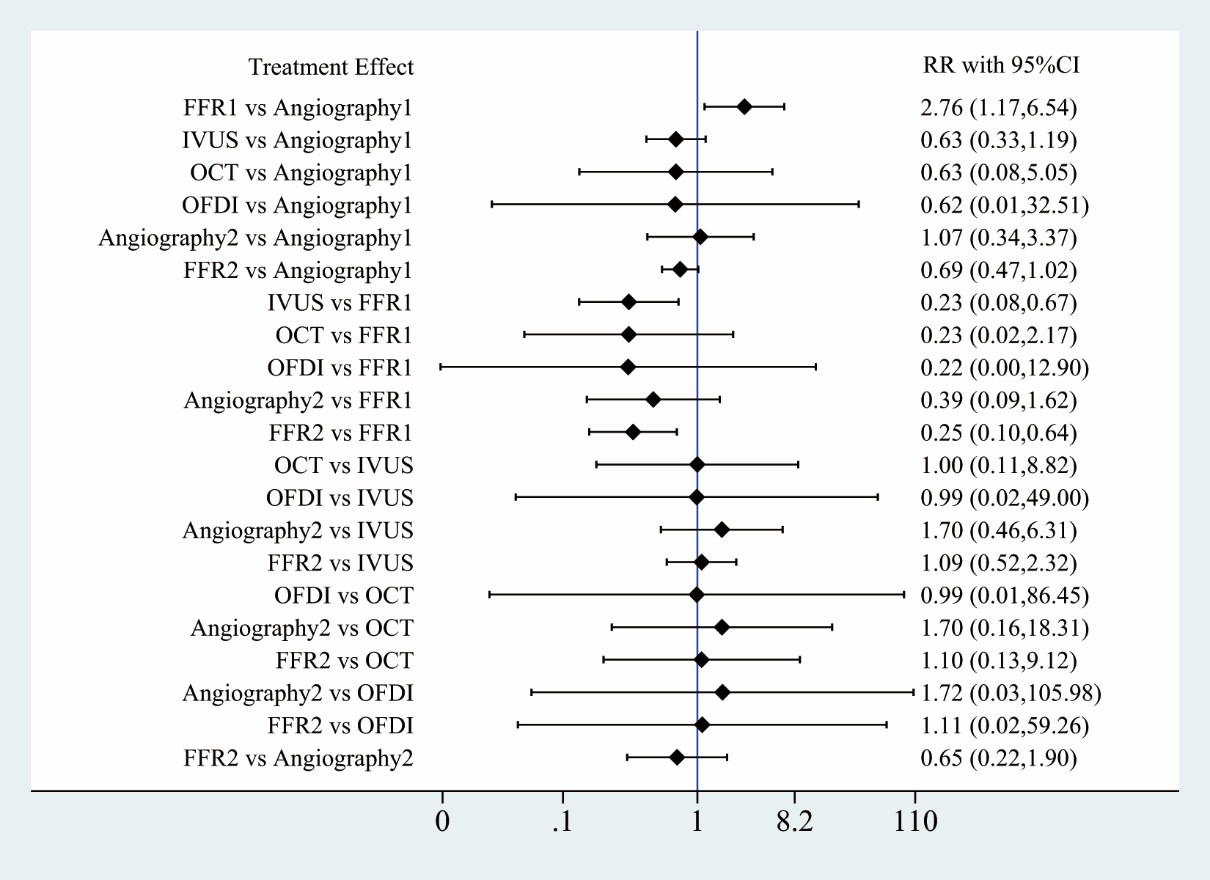


Supplementary Table 1. The search strategy in PubMed.

| #1 | Ultrasonography, Interventional [MeSH Terms] |
| --- | --- |
| #2 | Interventional Ultrasound [Title/Abstract] |
| #3 | Interventional Ultrasonography [Title/Abstract] |
| #4 | Intravascular Ultrasonography [Title/Abstract] |
| #5 | IVUS [Title/Abstract] |
| #6 | intra-vascular ultrasound [Title/Abstract] |
| #7 | intravascular ultrasound [Title/Abstract] |
| #8 | IVUS-guided [Title/Abstract] |
| #9 | ultrasound-guided [Title/Abstract] |
| #10 | #1 OR #2 OR #3 OR #4 OR #5 OR #6 OR #7 OR #8 OR #9 |
| #11 | Tomography, Optical Coherence [MeSH Terms] |
| #12 | OCT Tomography [Title/Abstract] |
| #13 | Optical Coherence Tomography [Title/Abstract] |
| #14 | OCT [Title/Abstract] |
| #15 | #11 OR #12 OR #13 OR #14 |
| #16 | OFDI [Title/Abstract] |
| #17 | optical frequency domain imaging [Title/Abstract] |
| #18 | #16 OR #17 |
| #19 | Fractional Flow Reserve, Myocardial [MeSH Terms] |
| #20 | Myocardial Fractional Flow Reserve [Title/Abstract] |
| #21 | functional-guided [Title/Abstract] |
| #22 | function-guided [Title/Abstract] |
| #23 | functional guided [Title/Abstract] |
| #24 | function guided [Title/Abstract] |
| #25 | FFR [Title/Abstract] |
| #26 | #19 OR #20 OR #21 OR #22 OR #23 OR #24 OR #25 |
| #27 | iFR [Title/Abstract] |
| #28 | instantaneous wave-free ratio [Title/Abstract] |
| #29 | #28 OR #29 |
| #30 | QFR [Title/Abstract] |
| #31 | Quantitative flow ratio [Title/Abstract] |
| #32 | #30 OR #31 |
| #33 | #10 OR #18 OR #26 OR #29 OR #32 |
| #34 | Myocardial Infarction [MeSH Terms] |
| #35 | MI [Title/Abstract] |
| #36 | ST Elevation Myocardial Infarction [MeSH Terms] |
| #37 | STEMI [Title/Abstract] |
| #38 | Non-ST Elevated Myocardial Infarction [MeSH Terms] |
| #39 | NSTEMI [Title/Abstract] |
| #40 | ACS [Title/Abstract] |
| #41 | acute coronary syndrome [MeSH Terms] |
| #42 | #34 OR #35 OR #36 OR #37 OR #38 OR #39 OR #40 OR #41 |
| #43 | randomized controlled trial [Title/Abstract] |
| #44 | controlled clinical trial [Title/Abstract |
| #45 | randomized [Title/Abstract] |
| #46 | randomly [Title/Abstract] |
| #47 | #43 OR #44 OR #45 OR #46 |
| #48 | #33 AND #42 AND #47 |

Supplementary Table 2. Definitions of the MACE and repeat revascularization.

| **Study** | **Major adverse cardiovascular events** | **Repeat revascularization** |
| --- | --- | --- |
| Jakabcˇin et al., 2009 | Composite of death from any cause, myocardial infarction, and TLR. | TLR |
| Ghani, et al., 2012 | Composite of death, nonfatal re-infarction and additional revascularization procedures. | Any additional revascularization procedures |
| Wang et al., 2014 | Composite of cardiac death, recurrent myocardial infarction, target vascular reconstruction, and intractable myocardial ischemia. | TVR |
| OCTACS, 2015 | NA | NA |
| DANAMI-3—PRIMULTI, 2015 | Composite of all-cause mortality, reinfarction, and ischemia-driven revascularization of lesions in non-infarct-related arteries. | Ischemia-driven revascularization |
| FAMOUS–NSTEMI, 2015 | Composite of cardiac death or hospitalization for myocardial infarction and heart failure after randomization. | NA |
| IVUS-XPL, 2015 | Composite of cardiac death, target lesion–related myocardial infarction, and ischemia-driven TLR | Ischemia-driven TLR |
| FAME, 2015 | Composite of death, myocardial infarction, and any repeat revascularization. | Any repeat revascularization |
| DOCTORS, 2016 | NA | TVR |
| Zhang, et al., 2016 | Composite of cardiovascular death, non-fatal myocardial infarction, and unplanned hospitalization for heart failure. | NA |
| ROBUST, 2017 | Composite of death, myocardial infarction, and TLR. | TLR |
| Compare-Acute, 2020 | Composite of all-cause mortality, non-fatal MI, any revascularization and cerebrovascular events. | NA |
| Fallesen et al., 2021 | NA | NA |
| FLOWER-MI, 2021 | Composite of death from any cause, nonfatal myocardial infarction, and unplanned hospitalization leading to urgent revascularization | Any revascularization |
| ULTIMATE, 2021 | Composite of cardiac death, target vessel MI, and clinically driven TVR | Clinically driven TVR |
| EROSION III, 2022 | NA | TLR |
| FAVOR III, 2022 | Composite of all-cause death, MI, and ischemia-driven revascularization. | Ischemia-driven revascularization |
| Barauskas et al., 2023 | NA | TLR |
| FRAME-AMI, 2023 | Composite of death, myocardial infarction, and repeat revascularization. | Any revascularization |
| Zhang, et al., 2023 | Combination of patient death due to any reason, nonfatal MI, and ischemia-induced revascularization. | Ischemia-driven revascularization. |
| ILUMIEN IV: OPTIMAL PCI, 2023 | Composite of death from cardiac causes, target-vessel myocardial infarction, and ischemia-driven target-vessel revascularization. | Ischemia-driven revascularization |
| OCTOBER, 2023 | Composite of death from a cardiac cause, target-lesion myocardial infarction, and ischemia-driven target-lesion revascularization. | Ischemia-driven TLR |
| OCTIVUS, 2023 | Composite of cardiac death, target vessel myocardial infarction, and clinically driven target vessel revascularization. | Any repeat revascularization. |
| FULL REVASC, 2024 | Composite of death from any cause, myocardial infarction, and unplanned revascularization. | Any planned or unplanned revascularization. |
| IVUS-ACS, 2024 | Composite cardiac death, target vessel myocardial infarction, or clinically driven target vessel revascularization. | Clinically driven target vessel revascularization. |
| OPINION ACS, 2024 | Composite of cardiac death, target vessel-related myocardial infarction, and ischemia-driven TVR. | TVR |
| Ullrich‑Daub, et al., 2024 | Composite of all-cause death, non-fatal myocardial infarction, unplanned hospitalization for angina and heart failure, unplanned revascularization. | Any revascularization |
| OCCUPI, 2024 | Composite of death from any cause, nonfatal myocardial infarction, and unplanned hospitalization leading to urgent revascularization. | Ischemia-driven target-vessel  Revascularization. |

Supplementary Table 3. Baseline characteristics.

| Study | Year | Randomized  treatments | Clinical presentation | Age | Sex  Male/Female | Revascularization strategies | Purpose of physiology and intravascular imaging guidance | Cut-off | Duration |
| --- | --- | --- | --- | --- | --- | --- | --- | --- | --- |
| Jakabcˇin et al. | 2009 | IVUS 105  Angiography 105 | ACS | 59.4±13  60.2±11 | 73/32  71/34 | Culprit-only  Culprit-only | Optimization | NA | 18m |
| Ghani, et al. | 2012 | FFR 79  Angiography 40 | STEMI | 62±10  61±11 | 64/16  33/8 | CR  Culprit-only | Decision-making | <0.75 | 36m |
| Wang et al. | 2014 | IVUS 38  Angiography 42 | STEMI | 56.4±9.4  53.7±11.8 | 23/15  28/14 | Culprit-only  Culprit-only | Decision-making | MLA <4.0 mm^2^ or plaque burden ≥70%, fibrous cap thickness of plaque <0.7 mm | 12m |
| OCTACS | 2015 | OCT 50  Angiography 50 | NSTEMI | 61.8±9.4  62.6±11.0 | 36/14  34/16 | Culprit-only  Culprit-only | Optimization | NA | 6m |
| DANAMI-3—PRIMULTI | 2015 | FFR 314  Angiography 313 | STEMI | 64 (37–94)  63 (34–92) | 251/63  255/58 | CR  Culprit-only | Decision-making | <0.80 | 27m |
| FAMOUS–NSTEMI | 2015 | FFR 176  Angiography 174 | NSTEMI | 62.3±11  61.6±11.1 | 133/43  127/37 | CR  CR | Decision-making | <0.80 | 12m |
| IVUS-XPL | 2015 | IVUS 342  Angiography 344 | ACS | NA | NA | Culprit-only  Culprit-only | Optimization | NA | 12m |
| FAME | 2015 | FFR 178  Angiography 150 | UA or NSTEMI | NA | NA | CR  CR | Decision-making | ≤ 0.80 | 60m |
| DOCTORS | 2016 | OCT 120  Angiography 120 | NSTEMI | 60.8±11.5  60.2±11.3 | 95/25  91/29 | Culprit-only  Culprit-only | Optimization | NA | 6m |
| Zhang, et al. | 2016 | FFR 110  Angiography 110 | NSTEMI | 70±3.7  70±3.7 | 75/35  78/32 | Culprit-only  Culprit-only | Decision-making | <0.80 | 12m |
| ROBUST | 2017 | OCT 105  Angiography 96 | STEMI | 57 (46–70)  59(47–72) | 87/28  83/13 | Culprit-only  Culprit-only | Optimization | NA | 9m |
| Compare-Acute | 2020 | FFR 290  Angiography 590 | STEMI | 62±10  61±10 | 233/62  450/140 | CR  Culprit-only | Decision-making | ≤ 0.80 | 36m |
| Fallesen et al. | 2021 | OCT 31  Angiography 32 | NSTEMI | 61.1±10.9  61.7±10.1 | 29/8  31/7 | Culprit-only  Culprit-only | Optimization | NA | 6m |
| FLOWER-MI | 2021 | FFR 586  Angiography 577 | STEMI | 62.5±11.0  61.9±11.4 | 498/88  468/109 | CR  CR | Decision-making | < 0.80 | 12m |
| ULTIMATE | 2021 | FFR 569  Angiography 567 | ACS | NA | NA | Culprit-only  Culprit-only | Optimization | NA | 12m |
| EROSION III | 2022 | FFR 112  Angiography 114 | STEMI | 54.5±11.2  56.4±10.4 | 89/23  91/23 | Culprit-only  Culprit-only | Optimization | NA | 12m |
| FAVOR III | 2022 | QFR 1213  Angiography 1215 | ACS | NA | NA | Culprit-only  Culprit-only | Decision-making | ≤ 0.80 | 24m |
| Barauskas et al. | 2023 | QFR 98  Angiography 100 | STEMI | 64.5±10.5  66.5±10.6 | 67/31  73/27 | CR  CR | Decision-making | < 0.80 | 12m |
| FRAME-AMI | 2023 | FFR 284  Angiography 278 | MI | 63.9±11.4  62.7±11.5 | 240/44  234/44 | CR  CR | Decision-making | < 0.80 | 42m |
| Zhang, et al. | 2023 | FFR 115  Angiography 114 | STEMI | 62.1±7.5  62.7±6.2 | 90/25  91/23 | CR  CR | Decision-making | ≤ 0.80 | 12m |
| ILUMIEN IV: OPTIMAL PCI | 2023 | OCT 727  Angiography 703 | ACS | NA | NA | Culprit-only  Culprit-only | Optimization | NA | 24m |
| OCTOBER | 2023 | OCT 270  Angiography 280 | ACS | NA | NA | Culprit-only  Culprit-only | Optimization | NA | 24m |
| OCTIVUS | 2023 | OCT 236  IVUS 234 | ACS | NA | NA | Culprit-only  Culprit-only | Optimization | NA | 12m |
| FULL REVASC | 2024 | FFR 764  Angiography 778 | MI | 65.0±10.3  65.7±10.6 | 601/163  576/202 | CR  Culprit-only | Decision-making | < 0.80 | 57.6m |
| IVUS-ACS | 2024 | IVUS 1753  Angiography 1752 | ACS | 62 (54–69)  63 (54–69) | 1285/468  1299/453 | Culprit-only  Culprit-only | Optimization | NA | 12m |
| OPINION ACS | 2024 | OFDI 70  IVUS 69 | ACS | 65.6±11.6  66.3±9.5 | 52/16  58/8 | Culprit-only  Culprit-only | Optimization | NA | 12m |
| Ullrich‑Daub, et al. | 2024 | QFR 101  Angiography 101 | ACS | 61 ± 10  66 ± 11 | 84/17  76/25 | CR  CR | Decision-making | < 0.80 | 12m |
| OCCUPI | 2024 | OCT 164  Angiography 168 | MI | NA | NA | Culprit-only  Culprit-only | Optimization | NA | 12m |

Supplementary Table 4. Assessment of between-study heterogeneity.

| **Outcome** | **Comparison** | **No. study** | **I^2^** |
| --- | --- | --- | --- |
| MACE | FFR vs Angiography | 10 | 64.09% |
|  | IVUS vs Angiography | 5 | 0 |
|  | OCT vs Angiography | 6 | 13.99% |
|  | QFR vs Angiography | 2 | 0 |
|  | OCT vs IVUS | 1 | NA |
|  | OFDI vs IVUS | 1 | NA |
| All-cause mortality | FFR vs Angiography | 9 | 5.41% |
|  | IVUS vs Angiography | 2 | 12.06% |
|  | OCT vs Angiography | 3 | 0 |
|  | QFR vs Angiography | 2 | 18.9% |
|  | OFDI vs IVUS | 1 | NA |
| Cardiac mortality | FFR vs Angiography | 6 | 15.21% |
|  | IVUS vs Angiography | 2 | 0 |
|  | OCT vs Angiography | 2 | 0 |
|  | OFDI vs IVUS | 1 | NA |
| MI | FFR vs Angiography | 9 | 45.91% |
|  | IVUS vs Angiography | 3 | 0 |
|  | OCT vs Angiography | 4 | 0 |
|  | OFDI vs IVUS | 1 | NA |
| Repeat revascularization | FFR vs Angiography | 7 | 72.95% |
|  | IVUS vs Angiography | 3 | 27.17% |
|  | OCT vs Angiography | 3 | 0 |
|  | QFR vs Angiography | 2 | 6.42% |
|  | OFDI vs IVUS | 1 | NA |
| Stent thrombosis | FFR vs Angiography | 5 | 54.68% |
|  | IVUS vs Angiography | 3 | 0 |
|  | OCT vs Angiography | 3 | 0 |
|  | OFDI vs IVUS | 1 | NA |

Supplementary Table 5. Network meta-analysis for all-cause mortality in decision-making or optimization cohorts.

| **Optimization** | **Decision-making** | | | | | |
| --- | --- | --- | --- | --- | --- | --- |
|  | **OFDI** | NA | NA | NA | NA | NA |
|  | NA | **QFR** | NA | NA | 0.26 (0.07,0.96) | 0.25 (0.07,0.92) |
|  | 0.09 (0.00,3.96) | NA | **OCT** | NA | NA | NA |
|  | 0.33 (0.01,7.93) | NA | 3.65 (0.47,28.18) | **IVUS** | NA | NA |
|  | NA | NA | NA | NA | **FFR** | 0.98 (0.78,1.24) |
|  | 0.20 (0.01,5.10) | NA | 2.22 (0.32,15.61) | 0.61 (0.33,1.11) | NA | **Angiography** |

Supplementary Table 6. Network meta-analysis for cardiac mortality in decision-making or optimization cohorts.

| **Optimization** | **Decision-making** | | | | |
| --- | --- | --- | --- | --- | --- |
|  | **OFDI** | NA | NA | NA | NA |
|  | 0.67 (0.01,44.96) | **OCT** | NA | NA | NA |
|  | 0.99 (0.02,49.00) | 1.47 (0.31,6.91) | **IVUS** | 1.53 (0.03,78.06) | 1.10 (0.02,54.53) |
|  | NA | NA | NA | **FFR** | 0.72 (0.43,1.21) |
|  | 0.44 (0.01,23.82) | 0.66 (0.17,2.51) | 0.45 (0.21,0.98) | NA | **Angiography** |

Supplementary Table 7. Network meta-analysis for MI in decision-making or optimization cohorts.

| **Optimization** | **Decision-making** | | | | |
| --- | --- | --- | --- | --- | --- |
|  | **OFDI** | NA | NA | NA | NA |
|  | 1.21 (0.03,44.14) | **OCT** | NA | NA | NA |
|  | 2.96 (0.12,71.38) | 2.45 (0.46,13.15) | **IVUS** | 0.30 (0.01,7.51) | 1.15 (0.84,1.56) |
|  | NA | NA | NA | **FFR** | 3.80 (0.15,95.63) |
|  | 1.90 (0.08,46.90) | 1.58 (0.31,8.12) | 0.64 (0.45,0.93) | NA | **Angiography** |

Supplementary Table 8. Network meta-analysis for repeat revascularization in decision-making or optimization cohorts.

| **Optimization** | **Decision-making** | | | | | |
| --- | --- | --- | --- | --- | --- | --- |
|  | **OFDI** | NA | NA | NA | NA | NA |
|  | NA | **QFR** | 0.22 (0.01,3.37) | 1.96 (0.07,53.02) | 0.68 (0.22,2.14) | 0.43 (0.15,1.28) |
|  | 2.26 (0.07,70.31) | NA | **OCT** | 9.07 (0.16,499.22) | 3.15 (0.25,40.29) | 2.00 (0.16,24.93) |
|  | 6.90 (0.32,148.38) | NA | 3.06 (0.65,14.43) | **IVUS** | 0.35 (0.02,7.98) | 0.22 (0.01,4.97) |
|  | NA | NA | NA | NA | **FFR** | 0.64 (0.44,0.92) |
|  | 3.60 (0.15,87.80) | NA | 1.59 (0.43,5.90) | 0.52 (0.21,1.27) | NA | **Angiography** |

Supplementary Table 9. Network meta-analysis for stent thrombosis in decision-making or optimization cohorts.

| **Optimization** | **Decision-making** | | | | |
| --- | --- | --- | --- | --- | --- |
|  | **OFDI** | NA | NA | NA | NA |
|  | 1.00 (0.01,87.66) | **OCT** | NA | NA | NA |
|  | 0.99 (0.02,49.00) | 0.99 (0.11,8.80) | **IVUS** | 0.55 (0.04,8.75) | 0.55 (0.04,7.79) |
|  | NA | NA | NA | **FFR** | 1.00 (0.45,2.19) |
|  | 0.63 (0.01,32.99) | 0.63 (0.08,5.05) | 0.64 (0.33,1.24) | NA | **Angiography** |
